# PKM2 promotes cell migration and inhibits autophagy by mediating PI3K/AKT activation and contributes to the malignant development of gastric cancer

**DOI:** 10.1038/s41598-017-03031-1

**Published:** 2017-06-06

**Authors:** Chao Wang, Jinling Jiang, Jun Ji, Qu Cai, Xuehua Chen, Yingyan Yu, Zhenggang Zhu, Jun Zhang

**Affiliations:** 1 Department of Oncology, Ruijin Hospital, Shanghai Jiao Tong University School of Medicine, No. 197 Ruijin Er Road, Shanghai, 200025 China; 2 Department of Surgery, Shanghai Key Laboratory of Gastric Neoplasms, Shanghai Institute of Digestive Surgery, Ruijin Hospital, Shanghai Jiao Tong University School of Medicine, No. 197 Ruijin Er Road, Shanghai, 200025 China

## Abstract

Pyruvate kinase M2 (PKM2) is a key kinase of glycolysis and is characteristic of all proliferating cells. The role of PKM2 in gastric cancer (GC) is still ambiguous and yet to be determined. To better understand the role of PKM2 in both the migration and invasion of GC, we measured the expression of PKM2 in GC cell lines using qRT-PCR and western blot. The prognostic value of PKM2 was analyzed by Immunohistochemistry in a cohort containing 88 GC patients. PKM2 was knocked down by the short hairpin RNA plasmid vector in NCI-N87 and BGC-823 cells, and the biological behavior and downstream signaling pathways were also investigated *in vitro*. Subcutaneous xenografts and pulmonary metastases models were constructed in nude mice to compare the differences in tumorgenesis and metastasis after Knockdown of PKM2. Our results obtained from *in vitro* cell biological behavior, *in vivo* tumorigenicity studies, and primary GC samples revealed an oncogenic role for PKM2 in GC. Furthermore, for those GC patients who received radical resection, PKM2 might serve as a novel prognostic biomarker and target which would allow for a brand new treatment strategy for GC in the clinical settings.

## Introduction

Gastric cancer (GC) is one of the most common malignancies worldwide and occurs at a highest frequency in Eastern Asia, especially in China^1^. According to statistics available for China in 2015, 679,100 new cases of gastric cancer were diagnosed and there were 498,000 reported deaths as a GC^2^. Due to the limited clinical approach in the early diagnosis and treatment of GC, the prognosis for GC patients is far from optimistic. A comprehensive understanding of the etiology and mechanisms of GC development will benefit the identification of novel targets associated with GC, which in turn would potentially lead to early detection, diagnosis and targeted treatment of this disease.

Pyruvate kinase isoform M2 (PKM2) is one of the isoenzymes of pyruvate kinase (PK), a key glycolytic enzyme which converts phosphoenolpyruvate (PEP) and adenosine diphosphate to pyruvate and adenosine triphosphate, as well as regulates glucose carbon flux into the cell^3^. PKM2 is expressed mostly in proliferating cells such as cancer cells, which is essential for shifting from regular cell metabolism to aerobic glycolysis. The latter provides selective growth advantages to cancer cells^4–6^. In addition to acting as a pyruvate kinase with a tetramer form, PKM2 plays a role as a protein kinase with a dimer form. The dimer conformation of PKM2 is mainly located in the nucleus and also stimulates the transcription factors, for example, it phosphorylates Tyr705 of STAT3 or it enhances STAT3 transcription activity^7^. Furthermore, nuclear PKM2 is directly bound to histone H3 and phosphorylated histone H3 at T11^8^ and it served as a transcriptional coactivator of aryl hydrocarbon receptor^9^. The above examples largely substantiate the fact that PKM2 promotes cell proliferation. Furthermore, overexpression of PKM2 accelerated oncogenic growth and autophagy inhibition in cancer cells^10^, while knockdown of PKM2 induced apoptosis and autophagy^11^. Aberrant PKM2 expression promotes malignant cellular transformation and is closely related to the clinical progression of solid tumors of the digestive system, including colorectal cancer, esophageal squamous cell carcinoma, oral cancer, biliary cancer, gastric cancer and hepatocellular carcinoma^12, 13^. Although proliferative activity and relative poor prognosis in GC have been shown to correlate directly with PKM2 expression, especially in signet ring cell gastric cancer^14, 15^, the exact role of PKM2 in GC and the mechanism by which it exerts its oncogenic role, is yet to be determined.

In this study, we investigated the expression of PKM2 in clinical GC samples and observed a correlation between PKM2 expression and poor clinical outcome of GC patients. Such a correlation was further confirmed in GC cell lines both *in vivo* and *in vitro*. Finally, we identified PKM2 as an upstream regulator of PI3K/AKT (phosphatidylinositol 3-kinase/AKT) oncogenic pathway in GC, which can mediate both migration and autophagy.

## Results

### Database analysis revealed that PKM2 is overexpressed in human cancers

PKM2 mRNA levels in human cancers were investigated using the datasets from the publicly available Oncomine database (www.oncomine.org). PKM2 mRNA expressions were found to be significantly elevated in human gastric cancer tissues compared to those in normal tissues using Cui and Wang datasets (Supplementary Fig. S1A)^16, 17^. In terms of Lauren’s classification, result from Ooi dataset indicated that PKM2 mRNA expression was significantly higher in intestinal-type as compared to diffuse-type and mix-type (Supplementary Fig. S1A)^18^. Besides, PKM2 mRNA levels were also elevated in colorectal cancer, esophagus cancer^19^, liver cancer^20^, lung cancer^21^, breast cancer^22^ and bladder cancer^23^ (Supplementary Fig. S1B and C). Therefore, high PKM2 expression is associated with various types of cancers in humans.

### PKM2 is overexpressed in gastric cancer tissues

**Figure 1:**
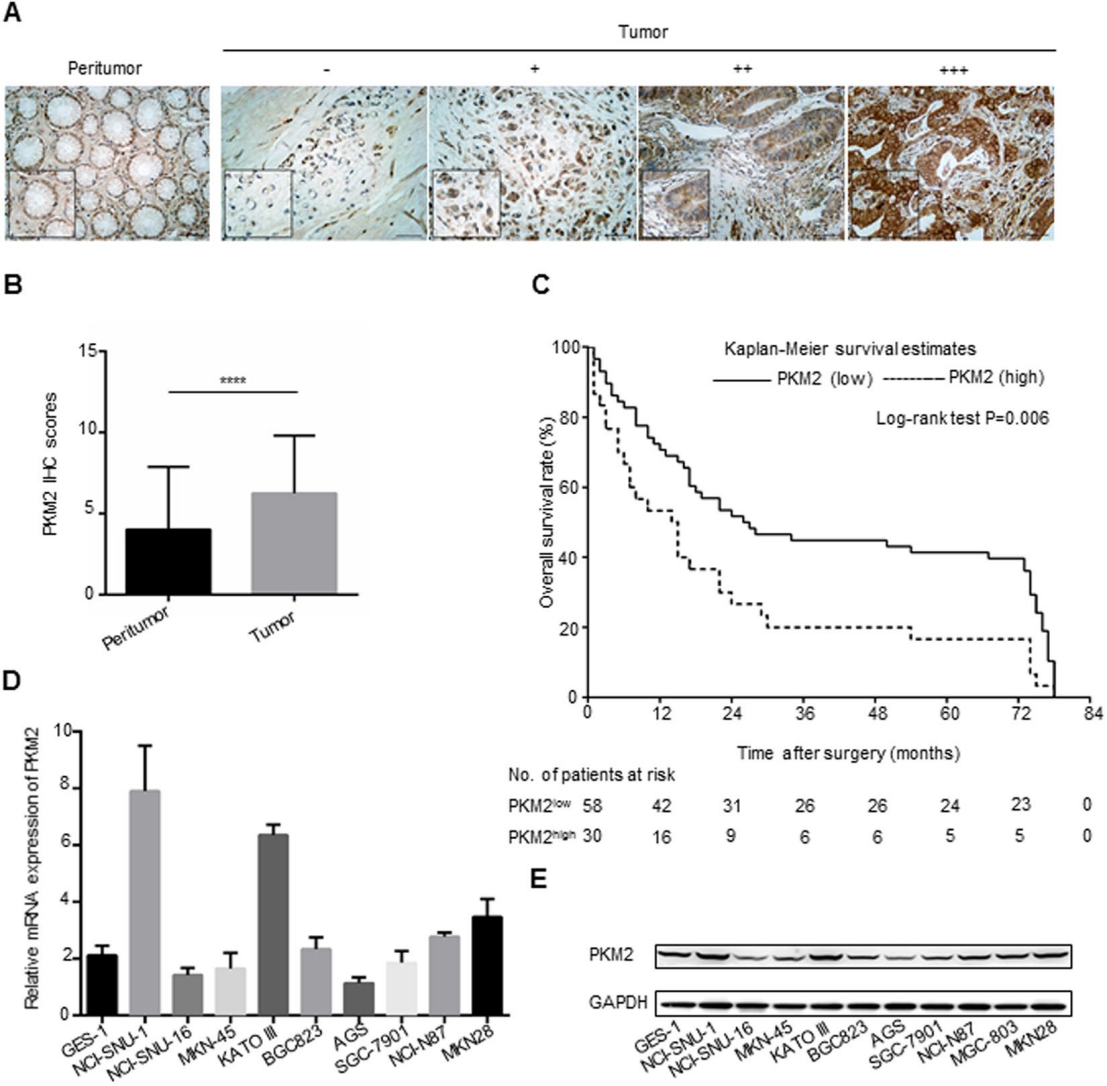
Expression and prognostic value of PKM2 in GC tissues (training cohort, n = 88) and expression of PKM2 in GC cell lines. (**A**) Representative images of PKM2 staining in peritumoral gastric tissues and tumor tissues of the different staining patterns are shown here and graded from 0 to +++. Scale bar, 200×, 100 μm. (**B**) Analysis of the IHC scores of PKM2 expression levels in human gastric cancer tissues compared with peritumoral gastric tissues. Error bar indicated standard deviation. (**C**) Kaplan-Meier analysis of overall survival for PKM2 protein expression, and the log-rank test identified PKM2 is significantly associated with cancer specific survival in GC patients. (**D**) Quantitative RT-PCR analysis of PKM2 mRNA levels in 10 GC cells and normal gastric epithelial cell line GES-1; GAPDH was used as a loading control. (**E**) Western blot analysis of PKM2 protein levels in 10 GC cells and normal gastric epithelial cell line GES-1. Full-length gels and blots are presented in the Supplementary files 2; GAPDH was used as a loading control. Data were shown as mean (±SD) from three independent experiments. ****P < 0.0001.

In order to measure the PKM2 protein expression levels in clinical gastric cancer tissues, IHC was performed in a cohort of 88 human gastric cancer tissues and matched non-tumor tissues. From the training cohort, representative IHC results are shown in Fig. 1A. PKM2 protein was predominantly located in the nucleus and cytoplasma, and was observed to have higher IHC scores in gastric cancer samples than in peritumor tissues, with the difference being statistically significant (P < 0.0001) (Fig. 1B).

### PKM2 expression correlates with poor prognosis and clinic-pathologic parameters in GC patients

The relationship between PKM2 expression and patients’ outcome was determined by Kaplan-Meier analysis. Results showed that high expression of PKM2 was closely correlated with worse overall survival (23 months versus 43 months, P = 0.006), moreover, we found that the 3-year and 5-year survival rates of the PKM2^low^ patients were significantly higher than those of the PKM2^high^ group (44.83% versus 20%, 41.38% versus 16.67%, respectively; Fig. 1C). The association between patients’ outcome and the PKM2 protein level was further analyzed using univariate and multivariate Cox regression models. In the univariate analysis, TNM staging (P = 0.005, HR = 1.916) and PKM2 expression (P = 0.031, HR = 1.873) were statistically significant risk factors affecting the overall survival (OS) of the patients. In the multivariate analysis, TNM staging (P = 0.006, HR = 2.02) and PKM2 expression (P = 0.037, HR = 1.715) were found to be statistically significant independent risk factors for OS of the patients (Table 1).

**Table 1:** Univariate and multivariate analyses of factors associated with survival in 88 cases of gastric cancer.

Variable	Univariable overall survival			Multivariable overall survival		
	Hazard Ratio	95% Confidence Limits	P Value	Hazard Ratio	95% Confidence Limits	P Value
Age(yrs)						
<65 (n = 39)	1					
≥65 (n = 49)	0.737	0.449–1.209	0.227			
Gender						
Men (n = 60)	1					
Women (n = 28)	0.871	0.518–1.465	0.603			
Tumor size (cm)						
<6.5 (n = 52)	1					
≥6.5 (n = 36)	1.46	0.683–1.93	0.149			
Lymph node metastasis						
Negative (n = 48)	1					
Positive (n = 40)	1.709	0.096–3.096	0.076			
Lymphatc invasion						
Negative (n = 71)	1					
Positive (n = 17)	1.191	0.656–2.161	0.566			
Tumor invasion						
T1 + T2 (n = 44)	1					
T3 + T4 (n = 44)	1.528	0.873–2.873	0.137			
TNM staging						
I + II (n = 58)	1			1		
III + IV (n = 30)	1.916	1.214–3.03	0.005	2.02	1.224–3.344	0.006*
Tumor location						
Gastric fundus (n = 17)	1					
Gastric corpus (n = 26)	1.291	0.646–2.582	0.47			
Pylorus (n = 45)	0.863	0.455–1.638	0.652			
Pkm2						
Low (n = 58)	1			1		
High (n = 30)	1.873	1.06–3.311	0.031	1.715	1.033–2.841	0.037*

*The difference is significant in multivariate analyses.

The correlation analysis between PKM2 expression in gastric cancer samples and clinicopathological parameters is summarized in Table 2. PKM2 expression was positively correlated with lymph node metastasis (P = 0.049), tumor invasion (P = 0.007) and TNM staging (P = 0.001). Other clinical characteristics, including age, gender, tumor size, lymphatic invasion and tumor location were not directly related to the PKM2 expression.

**Table 2:** Summary of the data on the expression of PKM2 in clinicopathological features of 88 cases of gastric cancer.

Category	No. patients (%)	PKM2		
		Negative	Positive	P value
Age(yrs)				
<65	39 (44.3)	25 (43.1)	14 (46.7)	
≥65	49 (55.7)	33 (56.9)	16 (53.3)	0.75
Gender				
Men	60 (68.2)	38 (65.5)	22 (73.3)	
Women	28 (31.8)	20 (34.5)	8 (26.7)	0.456
Tumor size (cm)				
<6.5	52 (59.1)	32 (55.2)	20 (66.7)	
≥6.5	36 (40.9)	26 (44.8)	10 (33.3)	0.299
Lymph node metastasis				
Negatie	48 (54.5)	36 (62.1)	12 (40.0)	
Positive	40 (45.5)	22 (37.9)	18 (60.0)	0.049*
Lymphatic invasion				
Negatie	71 (80.7)	47 (81.0)	24 (80.0)	
Positive	17 (19.3)	11 (19.0)	6 (20.0)	0.907
Tumor invasion				
T1 + T2	44 (50.0)	35 (60.3)	9 (30.0)	
T3 + T4	44 (50.0)	23 (39.7)	21 (70.0)	0.007*
TNM staging				
I + II	58 (65.9)	45 (77.6)	13 (43.3)	
III + IV	30 (34.1)	13 (22.4)	17 (56.7)	0.001*
Tumor location				
Gastric fundus	17 (19.3)	12 (20.7)	5 (16.7)	
Gastric corpus	26 (29.5)	17 (29.3)	9 (30.0)	
Pylorus	45 (51.1)	29 (50.0)	16 (53.3)	0.9

*The difference is significant in the chi-square test or Fisher’s exact test.

### PKM2 knockdown inhibits the tumor progression of GC *in vitro*

**Figure 2:**
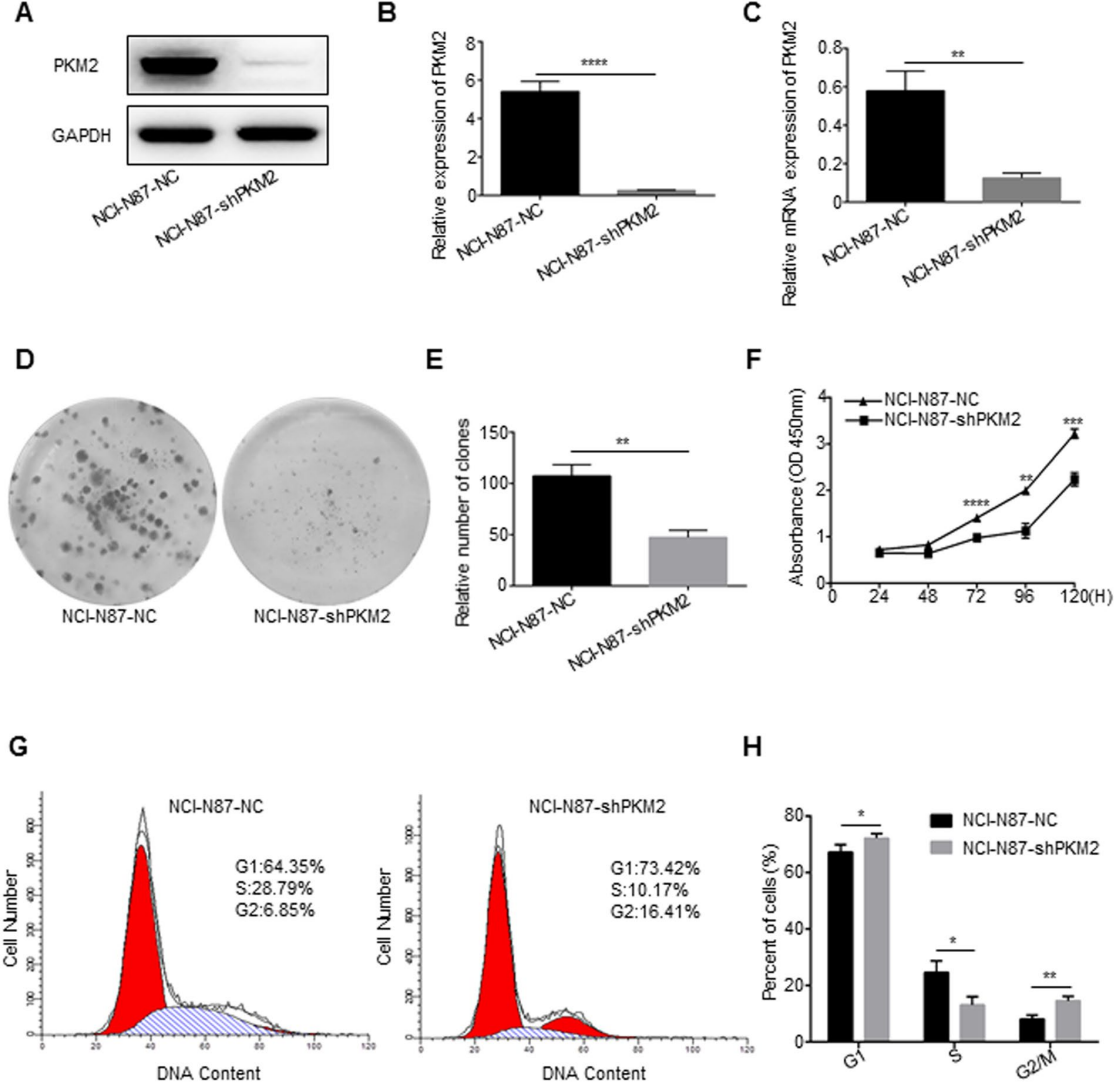
Effect of PKM2 knockdown on NCI-N87 cell growth *in vitro* and cell cycle. (**A**,**B** and **C**) PKM2 expression in NCI-N87 was modified by shRNA interference and verified with western blot and qRT-PCR, quantitative western blot analysis results obtained using densitometric analysis and the mRNA expression levels which were standardized according to GAPDH. Full-length gels and blots are presented in the Supplementary files 2. (**D** and **E**) Knockdown of PKM2 in NCI-N87 attenuated the ability of colony formation, data were shown as mean (±SD) from three independent experiments. (**F**) Knockdown of PKM2 in NCI-N87 attenuated the ability of cell proliferation which was detected by CCK-8 assay. (**G** and **H**) Knockdown of PKM2 attenuated the G1-S phase transition in NCI-N87, data were shown as mean (±SD) from three independent experiments. *P < 0.05. **P < 0.01. ***P < 0.001. ****P < 0.0001.

PKM2 mRNA and protein expression were also examined in a series of GC cell lines (NCI-N87, BGC-823, SGC-7901, AGS, SNU-1, SNU-16, MKN45, KATO III, MGC-803 and MKN28) and normal gastric mucosal cells GES-1 (Fig. 1D and E). We found that PKM2 expression level was relatively high in all 10 gastric cancer cell lines. However, the SNU-1 and KATO III cells had the highest expression of PKM2. We did not choose the above two cell lines for further studies due to the fact that they are not able to form the xenografts *in vivo*, thus, NCI-N87 and BGC-823 cells were judged to be appropriate ones. We used shRNA to generate PKM2-knockdown (PKM2-KD) cells to investigate the functions of PKM2. As shown in Fig. 2A–C, PKM2 shRNA treatment led to a significant decrease in PKM2 expression at the protein and mRNA levels (P < 0.0001 and P = 0.0017, respectively).

Firstly, we explored the effects of PKM2 downregulation on cell growth using the NCI-N87 cell line. In colony formation assay, the number of clones in NCI-N87-NC group was higher than that in the NCI-N87-shPKM2 group (P = 0.0014), and the sizes of clones formed in the NCI-N87-shPKM2 group was smaller than those in the control group (Fig. 2D and E). In proliferation assay, PKM2 shRNA treatment suppressed the growth of NCI-N87 cells (P < 0.0001; Fig. 2F); what we found reconfirmed that PKM2 promotes the growth of GC cells^15^.

Furthermore, the cell cycle assays revealed that the cell cycle was arrested in the G1 phase after knockdown of PKM2, with 73.42% of the NCI-N87-shPKM2 cells in G1 phase versus 64.35% of the control cells (P = 0.048; Fig. 2G and H). The result corresponded with the prior finding that showed that PKM2 regulated the G1-S phase transition in hepatocellular carcinoma^8^. PKM2 shRNA treatment had no effect on cellular apoptosis (Supplementary Fig. S4A).

**Figure 3:**
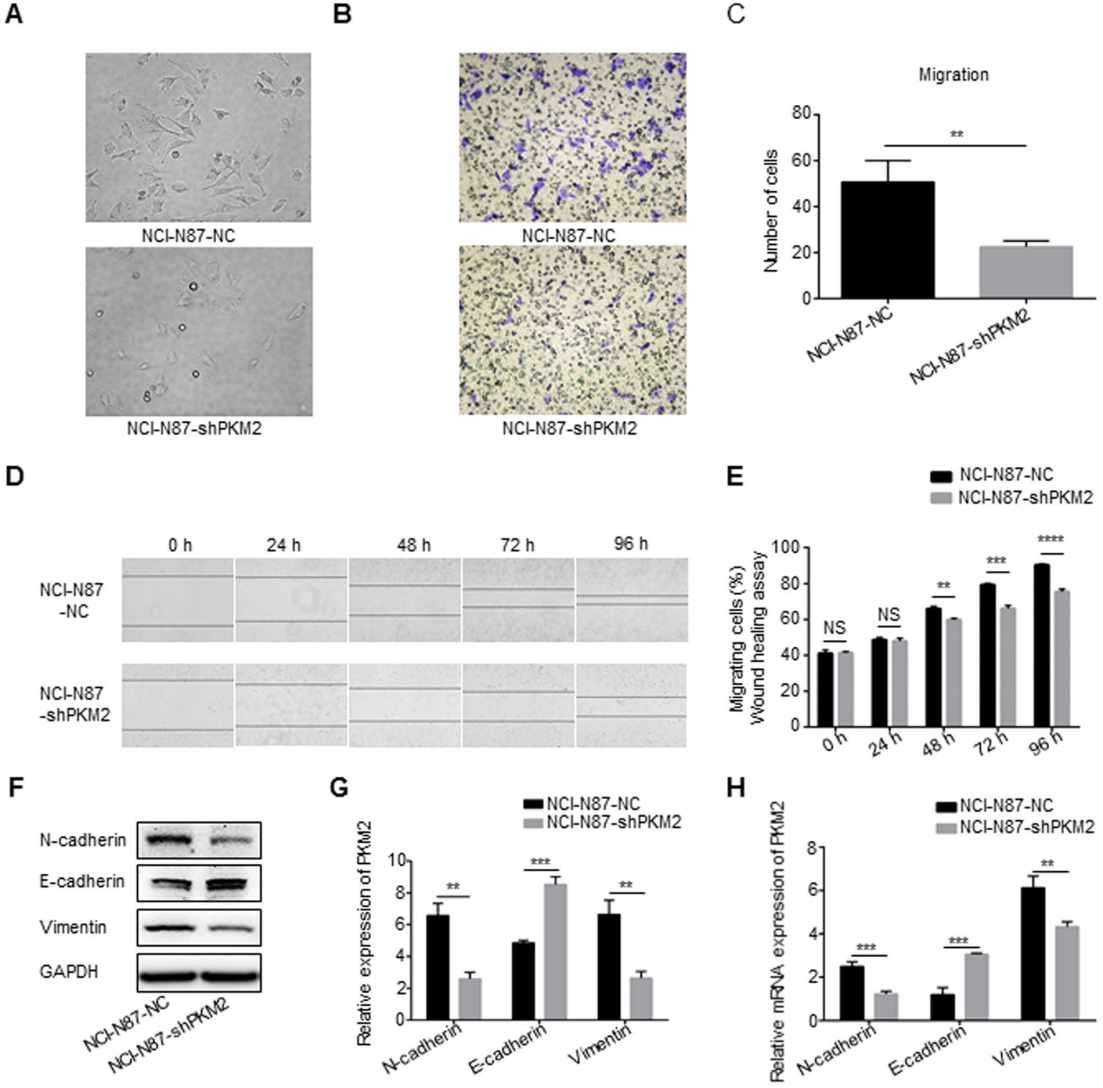
PKM2 knockdown inhibits the migration and mobility of NCI-N87 cell. (**A**) Knockdown of PKM2 reversed aggressive morphological characteristics in NCI-N87 cells (100× magnification). (**B** and **C**) Silencing of PKM2 expression led to lower migration rate in NCI-N87 cells. Representative photomicrographs showing that at the 24 h time point migrated cells were present (200× magnification). The data represent the mean (±SD) from three independent experiments. (**D** and **E**) Wound healing assays were performed in the NCI-N87 cells with different expression levels of PKM2 to investigate the cell mobility. Representative image of scratches at 0 h, 24 h, 48 h, 72 h and 96 h were shown. The results of the wound healing assays were also shown as graphs. The data represent the mean (±SD) from three independent experiments (40× magnification). (**F**,**G** and **H**) Expression levels of N-cadherin, E-cadherin and Vimentin in stably transducted NCI-N78 cells were analyzed by western blot and qRT-PCR, quantitative western blot analysis results obtained using densitometric analysis and the mRNA expression levels which were standardized according to GAPDH. Full-length gels and blots are presented in the Supplementary files 2. The data represent the mean (±SD) from three independent experiments. **P < 0.01. ***P < 0.001. ****P < 0.0001.

We, then, explored the effects of PKM2 downregulation on cell morphology using the NCI-N87 cell line. PKM2 shRNA treatment induced remarkable morphological changes, including elongated leading-trailing mesenchymal morphology to cobble-stone-like appearance and reduction of lamellipodia formation, indicating mesenchymal-epithelial transition (MET) (Fig. 3A). This observation suggested that low expression of PKM2 in GC cells might be correlated with the development of MET. Furthermore, to confirm that knockdown of PKM2 can not only cause MET but also alter cell migration, we performed the Boyden chamber assays and the wound-healing assays. *In vitro* migration assays showed that the number of migrated NCI-N87-NC cells was 50.7 ± 9.5, which was significantly higher than that of NCI-N87-shPKM2 cells (22.7 ± 2.5, P = 0.0077; Fig. 3B and C). The wound healing course of PKM2 knockdown groups were evidently slower than that of control ones (Fig. 3D and E). As is already known, epithelial-mesenchymal transition (EMT) is the switch from polarized epithelial cancer cells to contractile and motile mesenchymal cells during cancer progression and metastasis^24^. To further explore the role of PKM2 in the development of EMT, the typical EMT-related markers were also examined in NCI-N87 cells after treatment with PKM2 shRNA. Results from immunoblotting and qRT-PCR analyses showed that the epithelial marker E-cadherin was significantly upregulated (P = 0.0002 and P = 0.0006, respectively), while the mesenchymal markers N-cadherin and vimentin were dramatically downregulated (P = 0.0013, P = 0.0009, P = 0.0021 and P = 0.0061, respectively; Fig. 3F–H).

To further understand the role of PKM2 in tumor progression, we also knocked down PKM2 gene in BGC-823 cells with high PKM2 expression background (Supplementary Fig. S2A–C). As shown in Supplementary Fig. S2D and E, the colony formation ability of BGC-823-NC cells were higher than that of the BGC-823-shPKM2 cells (P = 0.0007). In proliferation assay, the proliferation ability of BGC-823-NC cells was also higher than that of the BGC-823-shPKM2 cells (P = 0.0004; Supplementary Fig. S2F). The PKM2 shRNA treatment arrested the cell cycle in the G1 phase, with 71.55% of the BGC-823-shPKM2 cells in G1 phase versus 51.52% of the BGC-823-NC cells (P = 0.0016; Supplementary Fig. S2G and H), while PKM2 knockdown did not induce cellular apoptosis (Supplementary Fig. S4B). As far as cell morphology, knockdown of PKM2 can also lead to mesenchymal-epithelial transition (MET) (Supplementary Fig. S3A). Furthermore, in the *in vitro* migration assays, the number of migrated BGC-823-NC cells was 124.3 ± 13.6, which was significantly higher than that of the BGC-823-shPKM2 cells (51.7 ± 5.1) (P = 0.001; Supplementary Fig. S3B and C). The knockdown of PKM2 obviously decreased the rate of wound healing as compared to the control cells (Supplementary Fig. S3D and E).

### PKM2 knockdown inhibits the tumor progression of GC *in vivo*

**Figure 4:**
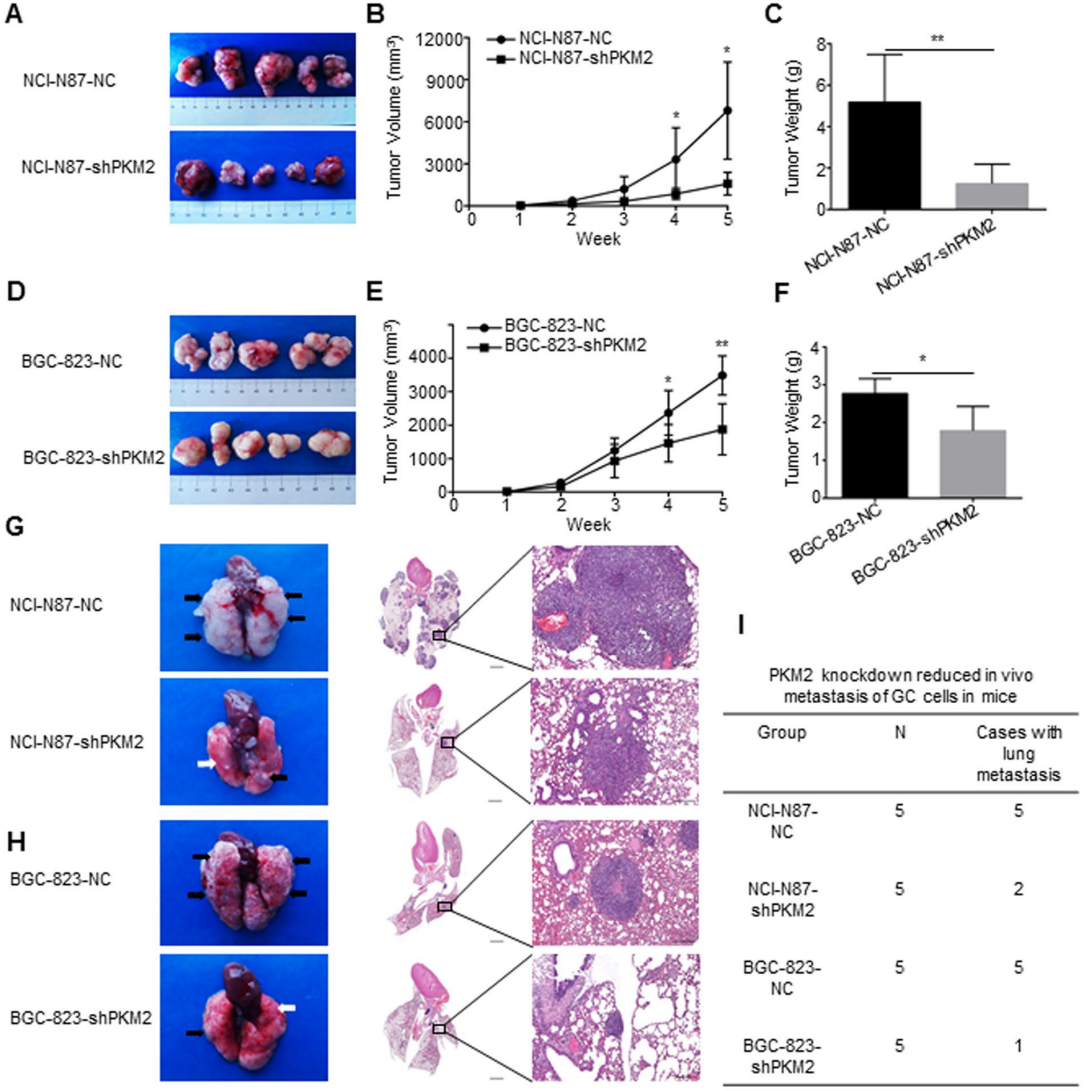
PKM2 knockdown reduces xenografts growth and lung metastasis of NCI-N87 and BGC-823 cells. (**A**) Tumor xenografts formed by implanted NCI-N87 cells with different expression levels of PKM2. (**B** and **C**) Tumor volume and weights of xenografts in subcutanea of nude mice were measured. Error bar indicated standard deviation (n = 5), Error bar indicated standard deviation (n = 5). (**D**) Tumor xenografts formed by implanted BGC-823 cells with different expression levels of PKM2. (**E** and **F**) Tumor volumes and weights of xenografts in subcutanea of nude mice were measured. Error bar indicated standard deviation (n = 5), Error bar indicated standard deviation (n = 5). (**G**) The images of lung metastasis and H&E stained sections of lung in the groups of NCI-N87 cells with different expression levels of PKM2 were shown. (**H**) The images of lung metastasis and H&E stained sections of lung in the groups of BGC-823 cells with different expression levels of PKM2 were shown. (**I**) Incidences of lung metastasis of each group were shown. n = 5 for each group. Black arrow stood for lung metastasis and white arrow stood for normal lung tissue. Scale bar, 6×, 2000 μm. 100×, 200 μm. *P < 0.05. **P < 0.01.

Knocking-down PKM2 significantly slowed down tumorigenesis in xenograft mice models of NCI-N87 cells and BGC-823 cells, respectively (P = 0.011 and P = 0.0056; Fig. 4A, B, D and E). Similar to the tumor size, the tumor weights of NCI-N87-NC and BGC-823-NC derived xenografts were 5.18 ± 2.30 g and 2.77 ± 0.39 g respectively, which were significantly heavier than those of xenografts originating from NCI-N87-shPKM2 cells and BGC-823-shPKM2 cells (1.25 ± 0.94 g and 1.78 ± 0.65 g respectively, P = 0.0077 and P = 0.0192; Fig. 4C and F). Moreover, the number and size of metastatic clusters in the NCI-N87-NC group and BGC-823-NC group exceeded those of the PKM2 knockdown groups under the vision of human eye and microscope (Fig. 4G and H), and the incidence of lung metastasis for NCI-N87-NC, NCI-N87-shPKM2, BGC-823-NC and BGC-823-shPKM2 cells were 100%, 40%, 100% and 20%, respectively (Fig. 4I).

**Figure 5:**
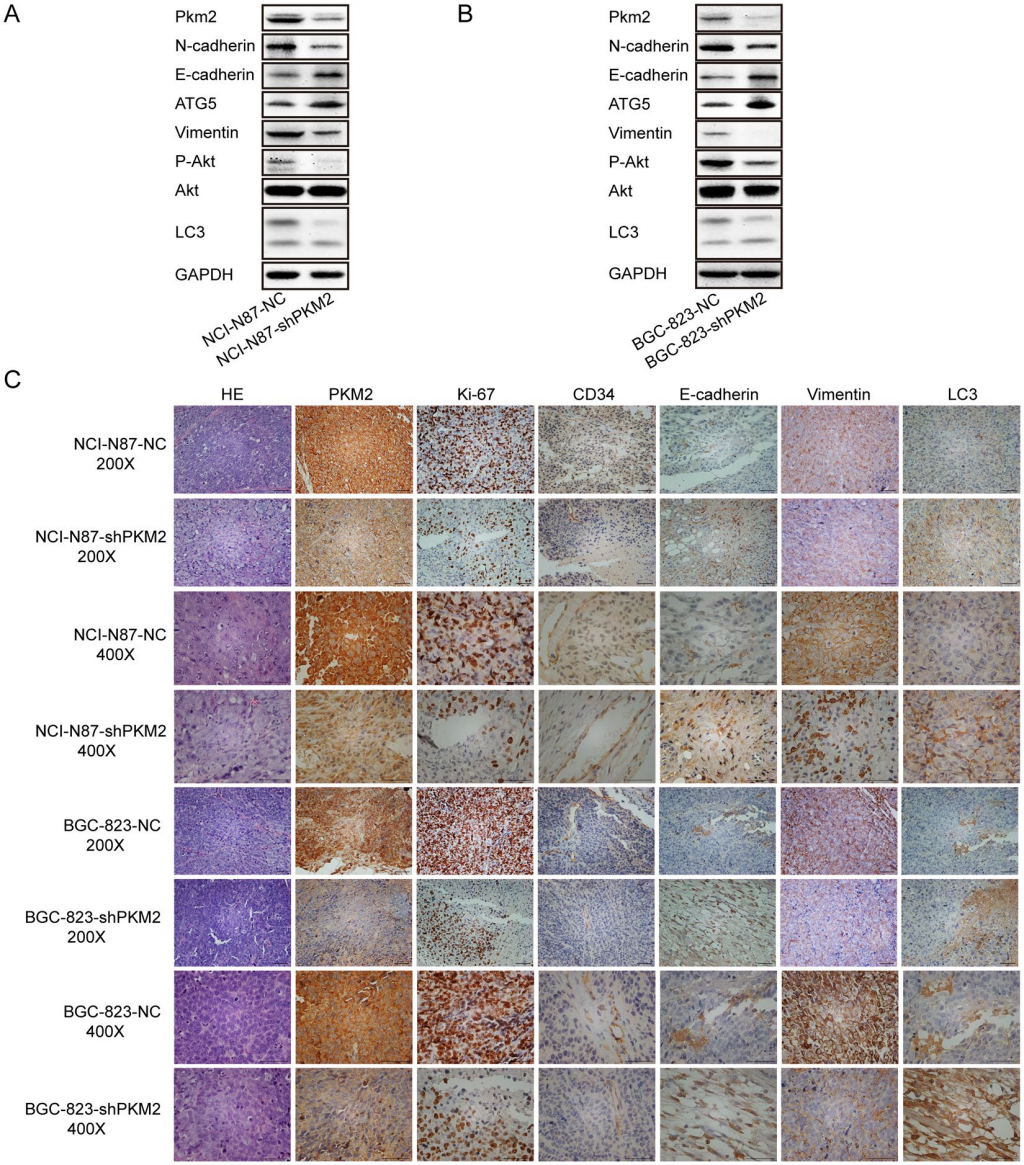
The effect of PKM2 knockdown on NCI-N87 cells and BGC-823 cells in a xenograft nude mice model. (**A** and **B**) Expression of N-cadherin, E-cadherin, ATG5, Vimentin, P-Akt, Akt and LC3 were mediated by PKM2 knockdown in the xenografts of nude mice. Full-length gels and blots are presented in the Supplementary files 2. (**C**) Representative photomicrographs from tumor serial sections stained with PKM2, Ki-67, E-cadherin, Vimentin, CD34 and LC3 by IHC. Scale bar, 200×, 100 μm, 400×, 50 μm.

PKM2 expression in xenografts was detected by both immunoblotting and IHC. In agreement with the level of protein *in vitro*, the intratumoral expression of PKM2, N-cadherin, E-cadherin and Vimentin in NCI-N87-NC-derived xenografts and BGC-823-NC-derived xenografts were markedly higher than those observed in NCI-N87-shPKM2-derived xenografts and BGC-823-shPKM2-derived xenografts by immunoblotting analysis, respectively (Fig. 5A and B). Similar results were observed in IHC assays, moreover, Ki-67 expression and microvessel density (MVD) were substantially decreased in the NCI-N87-shPKM2 and BGC-823-shPKM2 groups, as compared to those in the control groups (Fig. 5C), which were consistent with the role of PKM2 in pancreatic cancer impairing tumor growth and decreased blood vessel formation *in vivo* ^25^. Besides, the autophagy markers ATG5 and LC3 and P-Akt expression of all groups *in vivo* detected by immunoblotting or IHC were consistent with tendency of expression *in vitro* (Fig. 5A–C).

### PKM2 knockdown inhibits the PI3K/Akt signaling pathway

**Figure 6:**
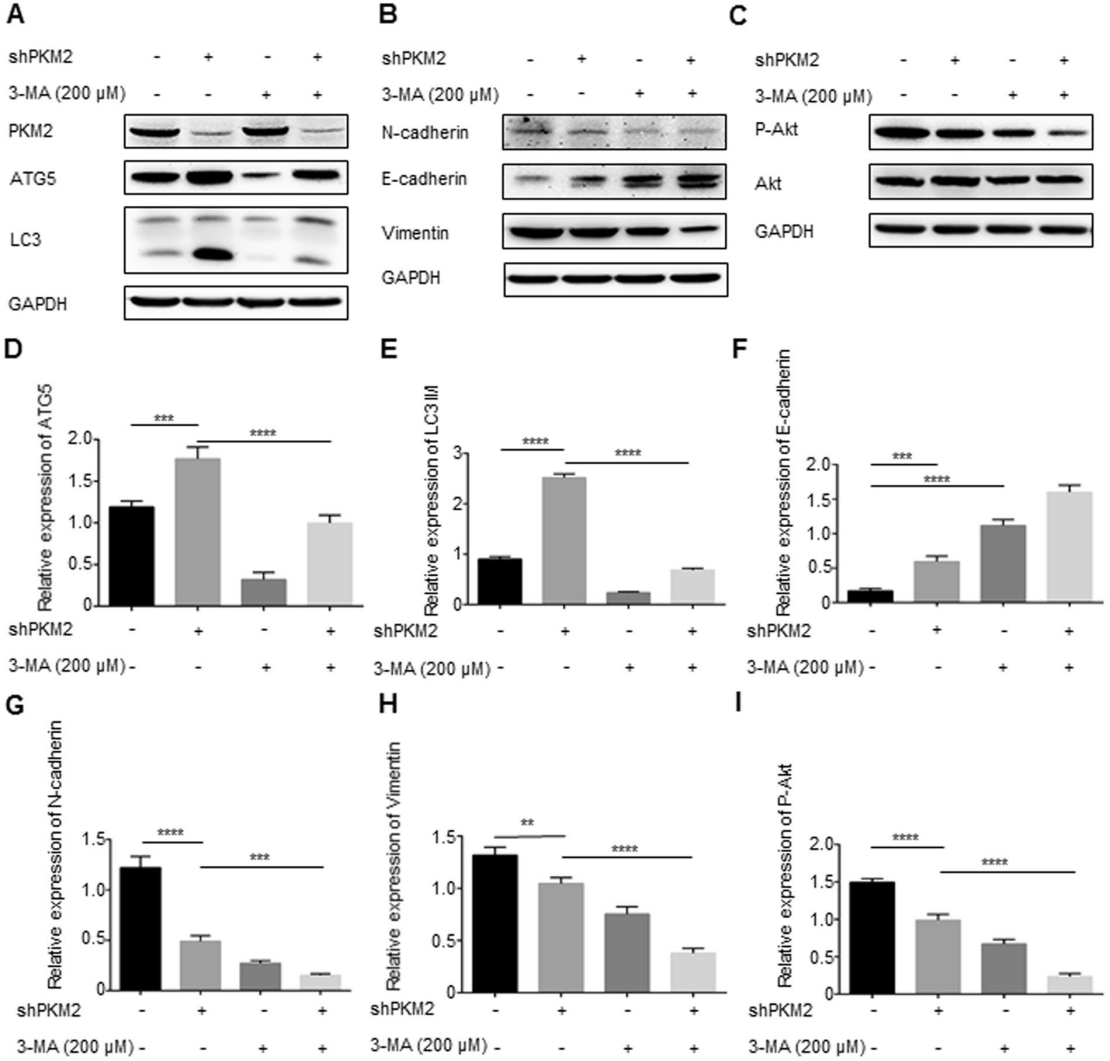
PKM2 mediates cell migration and autophagy via the PI3K/Akt signaling pathway. (**A**) Western blot analysis of PKM2, ATG5 and LC3 in NCI-N87 cells treated with knockdown of PKM2 in the presence of 3-MA (200 μM) for 24 h. (**B**) NCI-N87 cells were transducted with PKM2 shRNA and then treated with 200 μM 3-MA for 24 h, the expression levels of N-cadherin, E-cadherin and Vimentin were analyzed by western blot. (**C**) NCI-N87 cells were transducted with PKM2 shRNA and then treated with 200 μM 3-MA for 24 h. Cell lysates were prepared and subjected to western blot analysis using monoclonal anti-Akt and anti-phospho-Akt antibody. Full-length gels and blots are presented in the Supplementary files 2. (**D**–**I**) Relative expression of ATG5, LC3 II/I, E-cadherin, N-cadherin, Vimentin and P-Akt, and the quantitative western blot analysis results obtained using densitometric analysis, which were standardized according to GAPDH and calculated by ImageJ software (NIH). The data represent the mean (±SD) from three independent experiments. **P < 0.01. ***P < 0.001. ****P < 0.0001.

It has been reported that the overexpression of PKM2 can inhibit autophagy by activating mTORC1 in Hela, HEK293T and HCT116 cells^10^. We, therefore, tested whether PKM2 knockdown has an impact on autophagy in gastric cancer cells. The expression level of LC3 I/II and ATG5 were examined by immunoblotting to assess autophagy in the NCI-N87 and BGC-823 cells with or without PKM2 knockdown (Fig. 6A and Supplementary Fig. S3F). Downregulation of PKM2 resulted in the upregulation of the expression levels of ATG5 and LC3 I/II conversion (P < 0.001 and P < 0.0001, respectively), while use of 200 μM 3-MA (an autophagy inhibitor) for 24 h significantly reversed the overexpression of ATG5 and LC3 I/II conversion which induced by the PKM2 knockdown (P < 0.0001 and P < 0.0001, respectively; Fig. 6D and E).

As is already known, 3-MA can, not only, inhibit class III phosphoinositide 3-kinase (PI3K) but can also inhibit class I and II PI3Ks, which can be further corroborated by the inhibition of phosphorylation of Akt, the downstream target of class I PI3K^26^. After using 3-MA (200 μM) for 24 h, the expression level of the epithelial marker E-cadherin was markedly upregulated (P < 0.0001; Fig. 6B and F), while the mesenchymal markers N-cadherin and Vimentin were further downregulated in PKM2 shRNA groups (P < 0.001 and P < 0.0001, respectively; Fig. 6G and H, and Supplementary Fig. S3G). We, tentatively, put forward that PKM2 might mediate cell migration via PI3K/Akt signaling pathway.

We, therefore, investigated whether PKM2 shRNA treatment could down-regulate the phosphorylation level of Akt (P < 0.0001; Fig. 6C and I), and we further examined the role of PI3K/Akt signaling in the cell migration mediated by PKM2 (Fig. 6C and Supplementary Fig. S3H). All groups were treated with 3-MA (200 μM) for 24 hours, and the expression level of P-Akt in the PKM2 knockdown groups were further decreased (P < 0.0001; Fig. 6I). The results above indicate that PI3K/Akt signaling play a crucial role in PKM2 induced gastric cancer cell migration.

## Discussion

In the current study, we demonstrated that the expression of PKM2 is higher in GC tissues as compared to adjacent normal gastric tissues. We also illustrated the functions of PKM2 knockdown using *in vivo* and *in vitro* assays. The results of this study showed that PKM2 shRNA treatment inhibit GC cells proliferation and migration *in vitro*, arrest cells at the G1 stage of the cell cycle and restrain tumor growth and metastasis *in vivo*. To investigate whether PKM2 is associated with the clinical outcome of GC patients, we analyzed the expression of PKM2 in TMAs using IHC. We found that the expression of PKM2 in GC tissues is inversely correlated to patients’ OS. In addition to this, this study demonstrated that there is a remarkable positive correlation between the expression of PKM2 and lymph node metastasis, tumor invasion and TNM staging. It is, therefore, safe to assume that PKM2 might serve as a key oncogene which plays a central role in regulating tumor growth and metastasis in GC.

The functions of PKM2, as a key glycolytic enzyme, in cancer metabolism have been demonstrated unambiguously^3, 4, 27^, except for the metabolic functions of PKM2. It regulates gene expression or the cell cycle which are based on protein-kinase function^28, 29^. Accumulating evidence reveal that elevated PKM2 expression have been detected in various kinds of tumors^3, 13^, however, to the best of our knowledge, the relationship between PKM2 and GC remains unclear. One study reported that elevation of PKM2 is associated with poor prognosis for patients with signet ring cell gastric cancer^14^. Another basic study revealed that PKM2 maintains GC cell survival through the regulation of Bcl-xL expression^15^, although, a prior study indicated that PKM2 attenuates cell invasion and motility when E-cadherin is present^30^. These findings, however, still hold a certain degree of uncertainty. We also chose the BGC-823 cell to perform the transwell and wound healing assays and found that PKM2 knockdown inhibit cell migration and motility when E-cadherin is present. More importantly, the number and size of lung metastasis clusters formed by BGC-823-NC cells exceeded those of the BGC-823-shPKM2 group.

**Figure 7:**
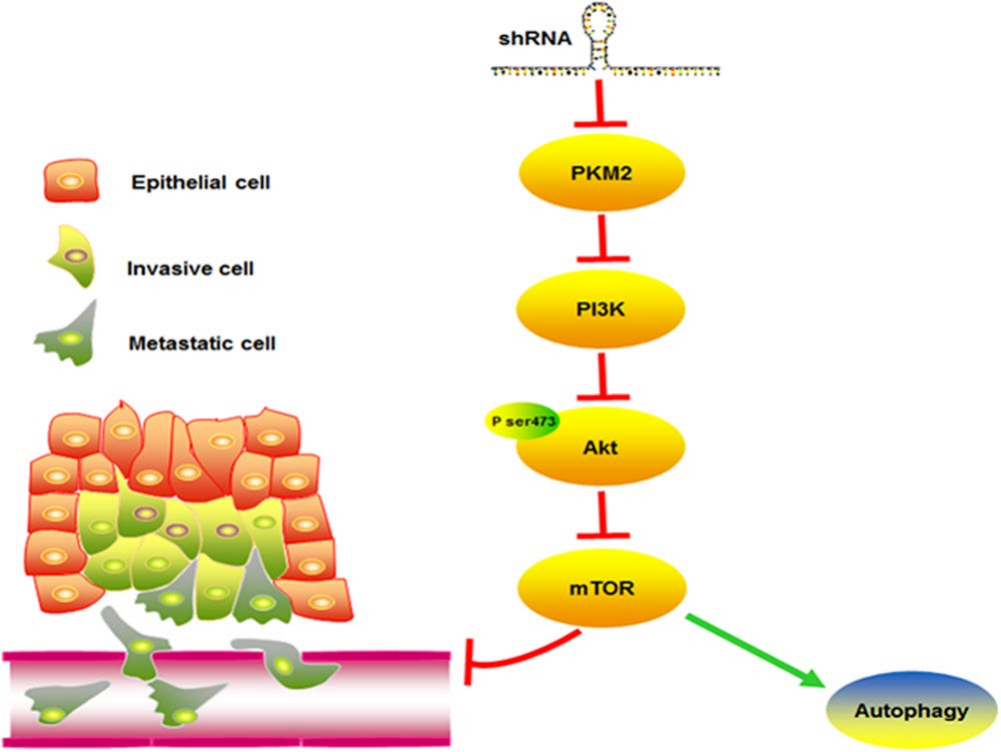
Schematic diagram of the effect of PKM2 on GC and the related pathways. Knockdown of PKM2 could inhibit the PI3K-Akt-mTOR signaling pathway, which induced the autophagy activation and reduced cancer cell migration in human gastric cancer.

Our results indicate that knockdown of PKM2 can attenuate the phosphorylation of Akt, and use of 3-MA (also known as PI3K inhibitor) can further decrease the phosphorylation levels of Akt in PKM2 knockdown groups. Meanwhile, the mesenchymal markers, N-cadherin and Vimentin were further downregulated in PKM2 shRNA groups after 3-MA treatment, which is similar to our findings. PI3K-Akt signaling was reported to also participate in PKM2-mediated migration in colon cancer cells^31^. Moreover, PI3K/Akt/mTOR pathway was shown to be frequently activated in GC and was directly linked to the progression of GC^32^. The mammalian target of rapamycin (mTOR) is downstream of PI3K-Akt signaling pathways and the activation of mTOR can contribute to autophagy inhibition^33^. As shown in a previous study, PKM2 overexpression can phosphorylate S202/203 of AKT1S1 and their phosphorylation activated mTORC1, which causes autophagy inhibition^10^. Therefore, knockdown of PKM2 might attenuate the PI3K-Akt-mTOR signaling pathway, which induces autophagy activation and reduces the ability of tumor migration in GC cells (Fig. 7).

As of now, studies have revealed that targeting PKM2 can, not only, mediate the biological activity of cancer cells, but also affect the effectiveness of therapies towards cancer, such as, in pancreatic cancer cells. The non-metabolic function of PKM2 was required for gemcitabine resistance, and the knockdown of PKM2 can induce enhanced apoptotic cell death during gemcitabine treatment via promotion of p53 activation. This may help to overcome the chemoresistance to gemcitabine in pancreatic cancer patients^34^. In addition to this, in lung cancer cells, PKM2 knockdown can similarly induce autophagy, and this autophagy protected the cells from apoptotic cell death which was also caused by the down-regulation of PKM2^11^. Silencing of PKM2 expression can contribute to radiosensitivity of non-small cell lung cancer (NSCLC)^35^, moreover, the combination treatment of cisplatin and RNA interference targeting PKM2 can significantly enhance the anti-tumor activity in A549 lung cancer xenograft model^36^. Silencing of PKM2 can restore cisplatin sensitivity in esophageal squamous cell carcinoma^37^, and increase the efficacy of docetaxel in A549 cell xenografts *in vivo* and *in vitro* assays^38, 39^. Inversely, PKM2 knockdown desensitized cervical cancer cells to cisplatin *in vitro* ^40^. Furthermore, high PKM2 expression level may be a negative predictive biomarker for platinum sensitivity in advanced NSCLC patients treated with platinum-based chemotherapy^41^.

Given the fact that autophagy ensures the survival of GC cells that are resistant to chemotherapy^42^, therefore, autophagy inhibition could potentially solve the problem of drug resistance, which is the major issue associated with poor chemotherapy responses and also poor prognosis^33^. Although, previously a study had revealed that 3-MA attenuates the invasion of cancer cells, independently of autophagy inhibition, through inhibition of type I and II PI3Ks and probably other molecules^26^, meanwhile, 3-MA could further reduce the mesenchymal markers N-cadherin, vimentin and enhance the epithelial marker E-cadherin which are mediated by the knockdown of PKM2 as has been confirmed in our study. We suppose that the autophagy caused by PKM2 knockdown could serve as the safeguard to protect cells from harmful factors. Therefore, one limitation of our study, was that we mainly focused on the role of PKM2 on GC tumor progression, the role of autophagy, which is caused by the knockdown of PKM2 on cancer cell will need to be investigated in future studies. Another limitation was the fact that 3-MA could further inhibit PI3K activity and attenuate the phosphorylation of Akt which is caused by downregulation of PKM2. The roles of 3-MA and knockdown of PKM2 toward autophagy were contradictory. It has already been well established that 3-MA attenuate autophagy via inhibiting the class III PI3K activity^26^, besides, the activation of class I PI3K could lead to activating the Akt-mTOR signaling pathway, which in turn inhibits autophagy^43^. Thus, we hypothesize that induction of autophagy by depleting PKM2 is due to the suppression of class I PI3K while sparing of class III PI3K. To determine whether this hypothesis is true and the detailed mechanisms governing the above mentioned phenomenon needs to be further investigated. Besides, the mechanism of PKM2 that regulate the cell-cycle progression of brain tumor cells have been manifested the fact that PKM2 interact with the spindle checkpoint Bub3 and phosphorylate Bub3 at Y207^29^, whether it exist in GC cells is worthy of further investigation, and the phenomena and experience acquired from the study are of critical significance to explore the potential mechanism of GC cells in our future study. Lastly, one drawback of our study is its retrospective nature, subsequent therapies including chemotherapy, targeted therapy and external radiotherapy of the patients with GC recurrence might potentially influence the OS results.

In conclusion, we demonstrated that knockdown of PKM2 can inhibit GC cell proliferation, G1-S phase transition, can, especially, attenuate GC cell migration *in vivo* and *in vitro*, per contra, promote the autophagy, which may depend on mediating the PI3K-Akt signaling. Furthermore, PKM2 expression would be a novel prognostic indicator for GC patients who received curative resection. Altogether, our experiments revealed the role of PKM2 in facilitating the malignant development of gastric cancer, suggesting that PKM2 might be a potential therapeutic target for treating gastric cancer.

## Materials and Methods

### Statement

In this study, all the cells and human tissues experiments were performed in according to the guildlines and regulations which provided by Shanghai Key Laboratory of Gastric Neoplasms, Shanghai Institute of Digestive Surgery, Ruijin Hospital, Shanghai Jiao Tong University School of Medicine. The animal use and care protocol was reviewed and approved by the Institutional Review of Committee for Animal Use of the Shanghai Jiao Tong University.

### Cell lines and culture

Human gastric cancer cell lines NCI-N87, BGC-823, SGC-7901, AGS, SNU-1, SNU-16, MKN45, KATO III, MGC-803 and MKN28, and normal GES-1 gastric mucosal cells were provided by Shanghai Institute of Digestive Surgery, Shanghai, People’s Republic of China. These cells were routinely maintained and cultured.

### Transduction and Clone Selection

shRNA constructed in pLKO.1-puro specifically targeting the human and PKM2 sequences were purchased from Shanghai Genepharma Co.Ltd, Shanghai, People’s Republic of China. The shRNA target sequences were as follows: AGGCAGAGGCUGCCAUCUA. Control cells were transducted with a control shRNA that did not match any known human coding cDNA. Stable knockdown clones were pooled and used for the experiments. Stably transducted clones were validated by immunoblotting for PKM2.

### Wound healing and transwell assay

Cells were plated in 6-well plates at a concentration of more than 90%. Wounds were scratched with 20 μL sterile pipette tips and suspended cells were washed with PBS buffer and then serum-free medium was used for cell culture. Micrographs were taken every 24 hours. All experiments were performed in triplicate.

1 × 10^5^ cells suspended in 200 μL serum-free medium were added to upper chambers of transwell (8 μM for 24-well plate, Millipore), and 600 μL full medium were added to lower chambers. After 24 hours, cells were fixed in formalin and stained with 0.1% crystal violet.

### *In vitro* proliferation and colony formation assay

Cells were plated into 96-well plates, with 500 cells per well and cultured overnight. CCK-8 (Dojindo Laboratories, Kumamoto, Japan) was, then, added and left to incubate at 37 °C for 2 h. The absorbance at 450 nm was detected by microplate spectrophotometer (BioTek, VT, USA) to calculate the number of vital cells in each well. The process lasted for 5 days, after which cell growth curves were drawn. All experiments were performed in triplicate. So as to determine the effects over a more extended period of time, 1000 cells were seeded in a 6-well plate in complete medium and incubated for 14 days at 37 °C and then fixed in 70% ethanol and finally stained with 0.1% crystal violet.

### Tissue Microarrays (TMAs) and Immunohistochemistrystaining (IHC)

TMAs were purchased from Shanghai biochip Co.Ltd, Shanghai, People’s Repulic of China. The histopathological diagnosis was according to the World Health Organization criteria. Tumor staging was based on the 6^th^ edition of the tumor-node-metastasis (TNM) classification of the International Union Against Cancer. Overall survival (OS) was defined as the time interval between the dates of surgery and death. For patients that passed away, the data were censored at the date of death and for surviving patient it was censored at the final follow-up.

Immunohistochemistry staining of paraffin sections were carried out on 4 μm-thick slices and confirmed to be a tumor by hematoxylin and eosin (HE) staining and then, followed by EnVision two-step procedure of Dako REAL^TM^Envision^TM^Detection System (Dako, Agilent Technologies, Ca, USA). After antigen retrieval, the slices were incubated overnight at 4 °C, with the primary antibodies including PKM2 (1:400, ABclonal), Ki-67 (1:50, Dako), LC3 (1:1000, CST), Vimentin (1:100, CST), E-cadherin (1:400, CST) and CD34 (1:200, Santa Cruz), respectively, followed by incubation with the HRP labeled secondary antibodies at 37 °C for half an hour and all slides were visualized with diaminobenzidine.

### Evaluation of immunohistochemical variables

PKM2 protein was localized in cell nucleus and cytoplasma, and stained as brownish granules. The expression status of PKM2 was scored using a 4-point scale (0~4) based on the number of positive cells and intensity of the staining under 5 random high-power fields. Score for percentage of positive cells were as follows: <5% (0), 5%~25% (1), 25%~50% (2), 50%~75% (3) and >75% (4). For intensity of staining, scores were as follows: no staining (0), light brown (1), brown (2) and dark brown (3). The final score for each specimen was determined by multiplying the staining intensity score with the score of stained cells percentage, scores of ≤ 2, 3~4, 6~8 and 9~12 were defined as negative (−), weak (+), moderate (++) and strong (+++) respectively. We defined 5 as the cut-off value to differentiate between the high and low expression of PKM2, GC patients with low or high expression were classified as PKM2^low^or PKM2^high^, respectively.

### Quantitative reverse transcription-polymerase chain reaction (qRT-PCR)

RNA was extracted by Trizol reagent method. Equal amounts of RNA were reverse transcribed into cDNA following protocol of Applied Biosystems. The primers used for all genes are listed in Supplementary Table S1. GAPDH was used as an internal control. Quantitative mRNA expression was measured by ABI Prism 7900HT sequence detection system (Applied Biosystems, CA, USA), and relative mRNA expression was calculated based on the Ct values, which were corrected for GAPDH expression according to the comparative Ct method^44^.

### Western blot analysis

Whole cell lysates were harvested using mammalian protein extraction reagent (Pierce, Rockford, IL, USA) mixed with protease inhibitor cocktail (Sigma-Aldrich, St. Louis, MO, USA). One hundred microgram protein samples were fractionated with 12.5% SDS-PAGE gel and transferred to PVDF membranes. The PVDF membranes were blocked with PBST buffer with 5% skim milk for 2 hours, then incubated at 4 °C with primary antibodies for PKM2 (1:1000, ABclonal Biotech Co., LTD, Woburn, Boston, USA), LC3 (1:1000, Cell signaling technology, Boston, USA), ATG5 (1:1000, Cell signaling technology, Boston, USA), AKT (1:1000, Cell signaling technology, Boston, USA), P-AKT (1:1000, Cell signaling technology, Boston, USA), Vimentin (1:1000, Cell signaling technology, Boston, USA), N-cadherin (1:1000, Cell signaling technology, Boston, USA) and E-cadherin (1:1000, Cell signaling technology, Boston, USA) overnight. GAPDH (1:2500, Santa Cruz Biotechnology, Santa Cruz, USA) was used as loading control. Secondary antibodies (1:15000, LI-COR, Nebraska, USA) and infrared imaging system (LI-COR Biosciences, Lincoln, USA) were used to visualize the protein bands.

### Flow cytometry analysis

For cell cycle analysis, 3 × 10^5^ cells were seeded in each well of the 6-well plates and cultivated for 24 h, then the cells were collected and fixed in 70% ethanol at 4 °C for at least 24 h and stained with 300 μL propidium iodide (PI) (BD Biosciences, Bedford, MA, USA), finally, the cell cycle distribution was analyzed by FACS (Becton-Dickinson, Bedford, MA, USA).

For apoptosis analysis, 3 × 10^5^ cells were seeded in each well of the 6-well plates and cultivated for 24 h, then the cells were collected and stained with Annexin V-FITC and propidium iodide (PI) (BD Biosciences Corporation, USA) and a flow cytometric analysis was performed using FACS (Becton-Dickinson, Bedford, MA, USA).

### *In vivo* tumorigenesis and metastasis

Twenty male Balb/c nude mice, 4–6 weeks of age and body weight ranging between 15 g and 20 g, were obtained from the Research Center of Experimental Medicine, Shanghai Jiaotong University School of Medicine Affiliated Ruijin Hospital. The animal use and care protocol was reviewed and approved by the Institutional Review of Committee for Animal Use of the Shanghai Jiao Tong University. These mice were randomly divided into 4 groups (5 mice in each group). One million NCI-N87/Vector, NCI-N87/shPKM2, BGC-823/Vector, and BGC-823/shPKM2 cells in 100 μL PBS were inoculated subcutaneously. Tumor nodules were measured every week after its length exceeded 2 mm, and the tumor volume was calculated using the following formula: V = (Width^2^× Length)/2, where V represents volume. Xenografts were collected at 4 weeks for immunohistochemistry staining and protein extraction.

### Pulmonary metastases models

Twenty male Balb/c nude mice (Research Center of Experimental Medicine, Shanghai Jiaotong University School of Medicine Affiliated Ruijin Hospital) were randomly divided into 4 groups (5 mice in each group). One million NCI-N87/Vector, NCI-N87/shPKM2, BGC-823/Vector, and BGC-823/shPKM2 cells in 100 μL PBS were, respectively, injected through the tail vein. One mouse from each group was sacrificed at 4 weeks after injection to evaluate metastasis formation. Eight weeks later, all mice were sacrificed. Pulmonary metastases were checked by gross specimens and microscopy.

### Statistical analysis

The chi-square test or Fisher’s exact test was used for categorical variable comparison. Overall survival of the patients in the groups of high and low PKM2 expression was calculated with the Kaplan-Meier method and differences between the survival curves were tested using the log-rank test. Univariate and multivariate survival analyses were performed using the Cox proportional hazards regression model. For the multivariate model, we used 0.20 as the cut-off P-value to select the analyzed factors from the univariate analysis data. A forward stepwise Cox regression model was also used to find independent prognostic factors. Independent-samples *t-*test and one-way analysis of variance (ANOVA) were used in quantitative data analysis. Data were expressed as mean and standard deviation ($\overline{x}\pm$S), and all the statistics analyses were performed using SPSS19.0 for Macintosh (SPSS Inc.; Chicago, IL, USA). Two-tailed p-value less than 0.05 were considered as statistically significant.

## Electronic supplementary material

Supplementary information
