# Supplementary material for: PKM2 promotes cell migration and inhibits autophagy by mediating PI3K/AKT activation and contributes to the malignant development of gastric cancer
Source: Sci Rep. 2017 Jun 6;7:2886. doi: 10.1038/s41598-017-03031-1 (PMC5460252; doi:10.1038/s41598-017-03031-1)
Supplement: Supplementary file 1 — Supplementary information [file 41598_2017_3031_MOESM1_ESM.pdf]

PKM2 promotes cell migration and inhibits autophagy by mediating PI3K/AKT activation and contributes to the malignant development of gastric cancer

Chao Wang [1 #], Jinling Jiang [1 #], Jun Ji[2], Qu Cai[2], Xuehua Chen[2], Yingyan Yu[2], Zhenggang Zhu[1, 2], Jun Zhang[1] *

Supplementary Table S1

Sequence of primers for qRT-PCR

Gene	Forward primer (5'-3')	Reverse primer (5'-3')
PKM2	ACAGGAAGCCTCGTAAGGTC	CAGTCCCGGCTTCACTATGG
Vimentin	AACTTAGGGGCGCTCTTGTCCC	CGCGCTGCTAGTTCTCAGTGCT
E-cadherin	CCCACCACGTACAAGGGTC	CTGGGGTATTGGGGGCATC
N-cadherin	GAGGAGTCAGTGAAGGAGTCA	GGCAAGTTGATTGGAGGGATG
GAPDH	AATGGGCAGCCGTTAGGAAA	GCCCAATACGACCAAATCAGAG

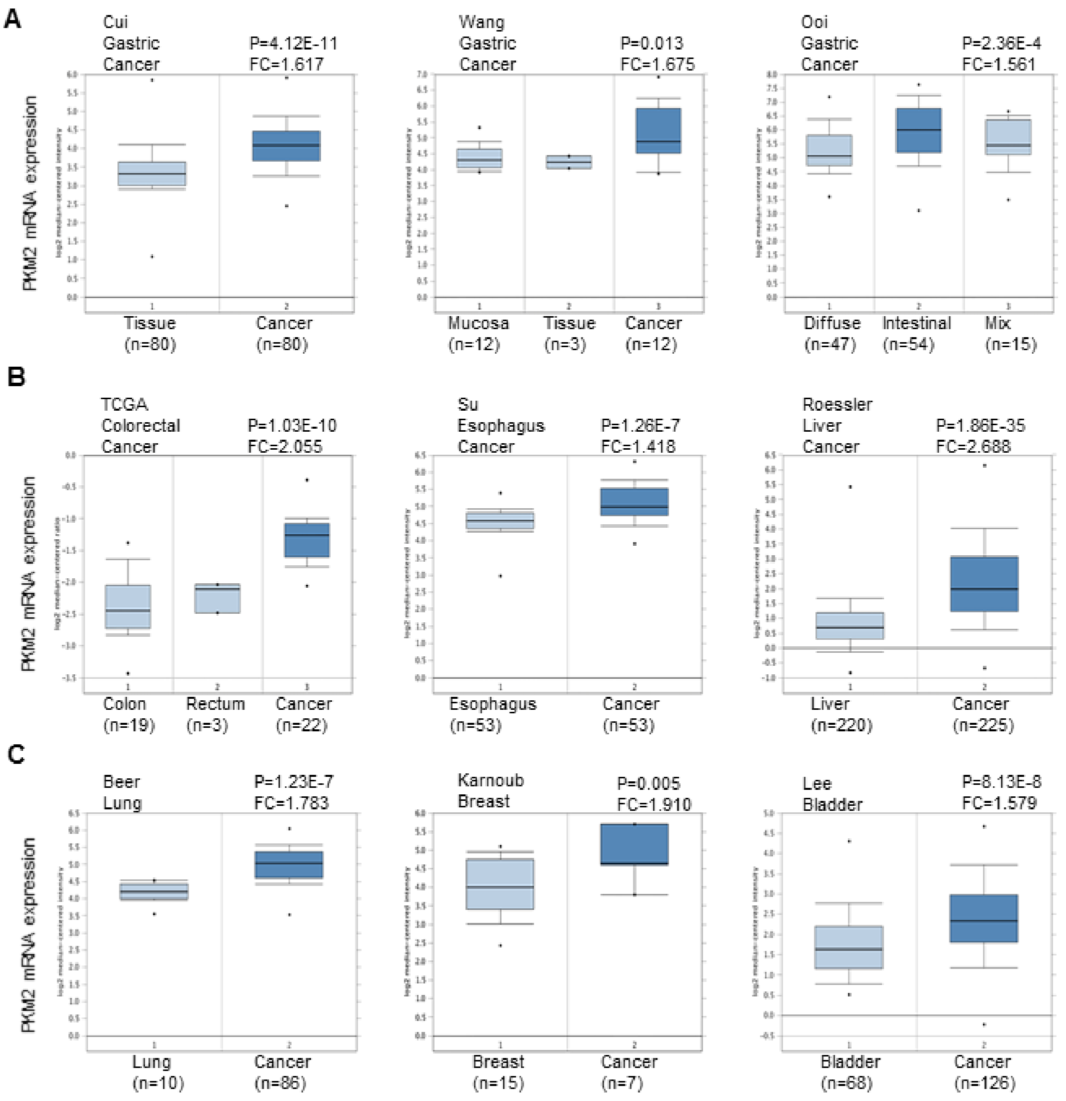

Supplementary Fig. S1. PKM2 mRNA expression in human cancers using the Oncomine database. A: PKM2 mRNA expression in human gastric cancer tissues including Lauren's classification and normal gastric tissues. B and C: PKM2 mRNA expression in human normal/cancer tissues (colorectum, esophagus, liver, lung, breast and bladder). The boxed part represent the 25th through 75th percentiles, the whiskers stand for the 10th and 90th percentiles, the horizontal lines show the medians, and the asterisks represent the end of the ranges.

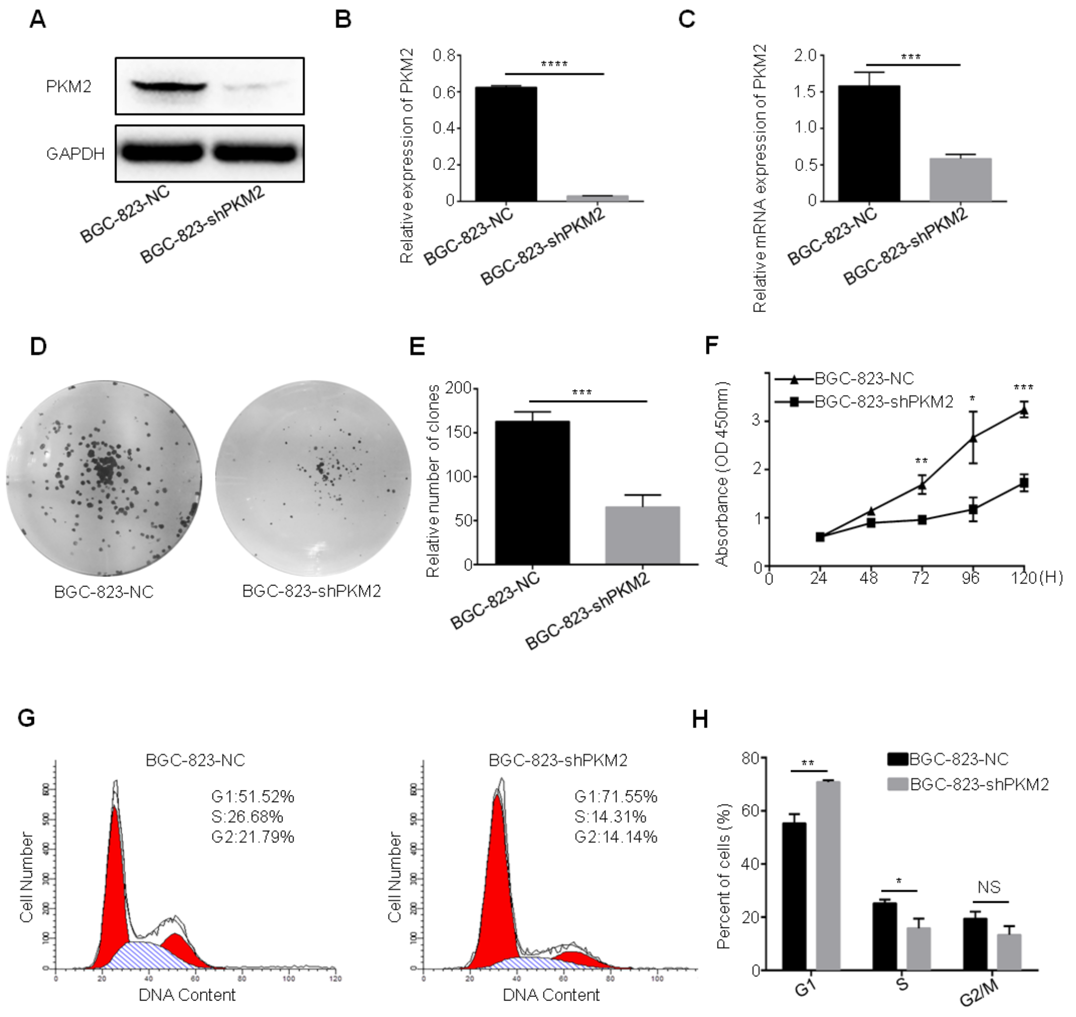

Supplementary Fig. S2. Effect of PKM2 knockdown on BGC-823 cell growth *in vitro* and cell cycle. A, B and C: PKM2 expression in BGC-823 was modified by shRNA interference and verified with western blot and RT-qPCR, quantitative western blot analysis results obtained using densitometric analysis and the mRNA expression levels which were standardized according to GAPDH. D and E: Knockdown of PKM2 in BGC-823 attenuated the ability of colony formation, data were shown as mean (±SD) from three independent experiments. F: Knockdown of PKM2 in BGC-823 attenuated the ability of cell proliferation which was detected by CCK-8 assay. G and H: Knockdown of PKM2 attenuated the G1-S phase transition in BGC-823 cells, data were shown as mean (±SD) from three independent experiments. *P < 0.05. **P < 0.01. ***P <0.001. ****P < 0.0001.

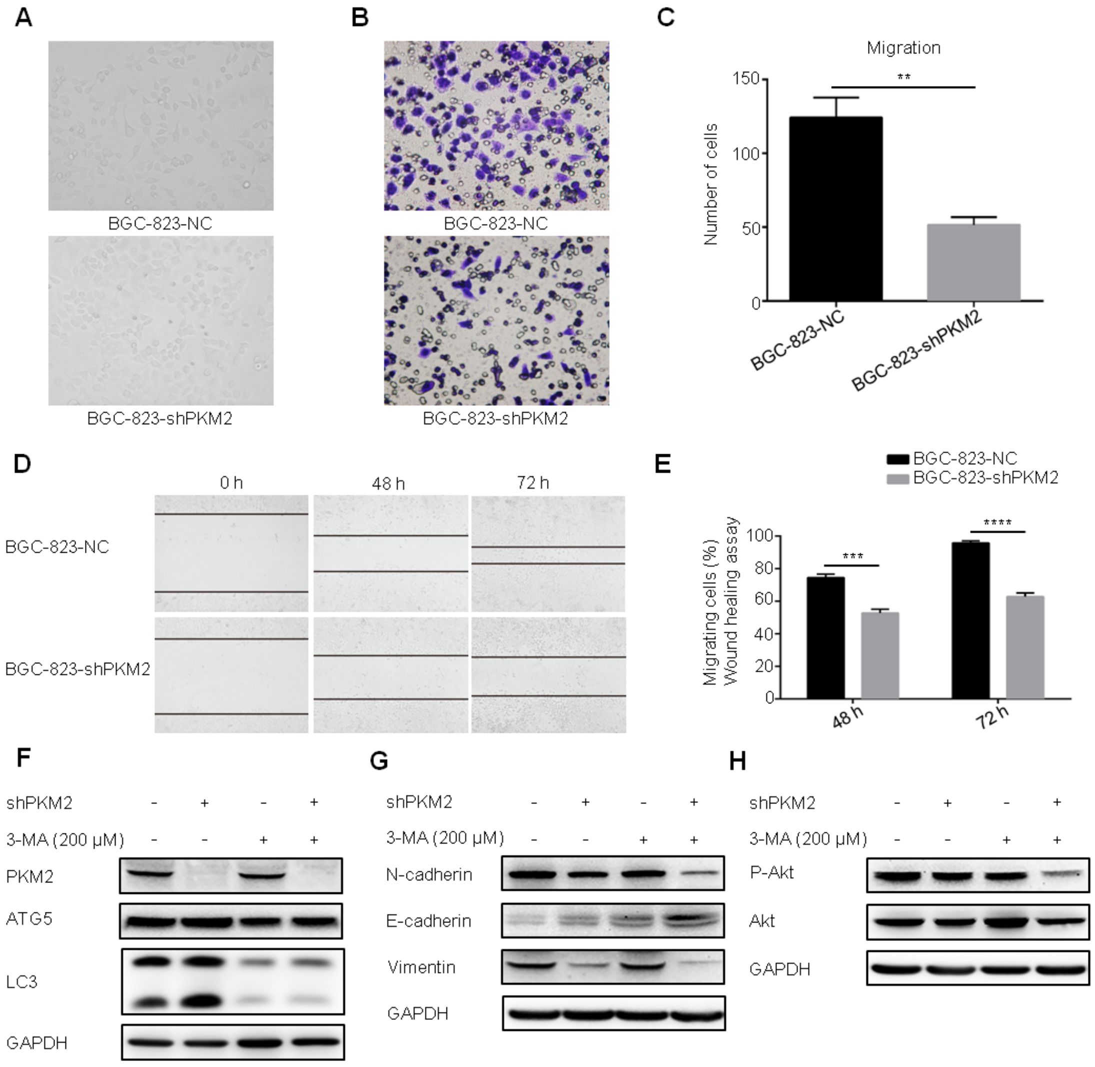

Supplementary Fig. S3. PKM2 mediates cell migration and autophagy via PI3K/Akt signaling pathway. A: Knockdown of PKM2 reversed aggressive morphological characteristics in BGC-823 cells (100× magnification). B and C: Silencing of PKM2 expression led to lower migration rate in BGC-823 cells. Representative photomicrographs of migrated cells were present (200× magnification). The data represent the mean (±SD) from three independent experiments. D and E: Wound healing assays were performed in the BGC-823 stably transducted cells to investigate the cell mobility. Representative image of scratches at 0 h, 48 h and 72 h were shown (40× magnification). The results of the wound healing assays were also shown as graphs. The data represent the mean (± SD) from three independent experiments. F: Western blot analysis of PKM2, ATG5 and LC3 in BGC-823 cells treated with knockdown of PKM2 in the presence of 3-MA (200 μM) for 24 h. G: BGC-823 cells were transducted with PKM2 shRNA, and then treated with 200 μM 3-MA for 24 h. Expression levels of N-cadherin, E-cadherin and Vimentin were analyzed by western blot. H: BGC-823 cells were transducted with PKM2 shRNA, and treated with 200 μM 3-MA for 24 h. Cell lysates were prepared and subjected to western blot analysis using monoclonal anti-Akt and anti-phospho-Akt antibody. **P < 0.01. ***P <0.001.

****P < 0.0001.

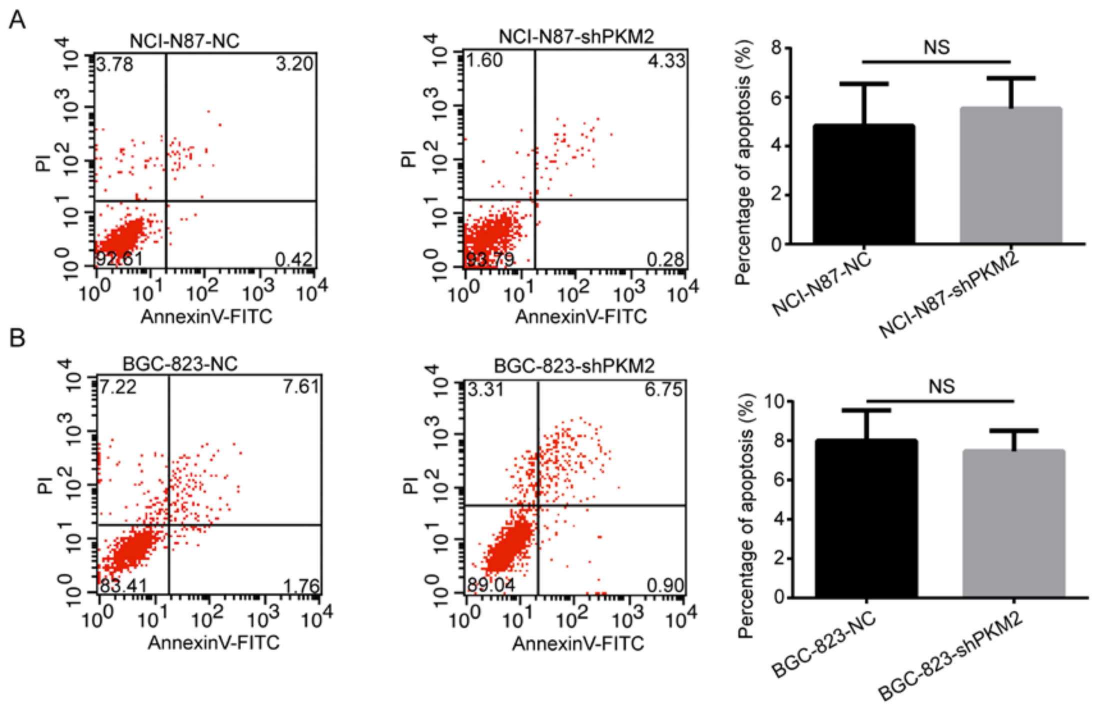

Supplementary Fig. S4. Knockdown of PKM2 have no influence on the apoptosis of cells. A: The apoptosis of NCI-N87 cells with different level of PKM2 were analyzed by flow cytometry for annexinV-FITC/PI assay. B: The apoptosis of BGC-823 cells with different level of PKM2 were analyzed by flow cytometry for annexinV-FITC/PI assay

Fig. 1B the PKM2 IHC scores of peritumor and tumor tissues (88 cases)

No.	peritumor	tumor
1	1	4
2	1	1
3	1	4
4	1	3
5	0	4
6	6	4
7	0	4
8	1	4
9	12	12
10	1	4
11	1	4
12	1	4
13	6	4
14	9	12
15	1	4
16	9	12
17	8	12
18	2	4
19	9	4
20	2	4
21	4	4
22	12	12
23	2	4
24	6	4
25	9	9
26	4	4
27	4	4
28	2	4
29	9	12
30	0	3
31	1	4
32	9	3
33	9	12
34	1	4
35	1	9
36	2	4
37	12	12
38	4	4
39	1	4
40	8	12
41	0	4

42	1	4
43	1	4
44	6	12
45	2	4
46	8	12
47	1	4
48	1	4
49	9	12
50	1	4
51	1	4
52	8	12
53	1	4
54	1	4
55	1	4
56	1	3
57	1	4
58	1	4
59	2	4
60	1	3
61	1	4
62	9	12
63	12	4
64	9	12
65	1	4
66	0	4
67	8	6
68	12	12
69	1	4
70	12	12
71	1	4
72	1	4
73	9	9
74	1	9
75	2	12
76	6	4
77	6	4
78	2	4
79	9	12
80	6	4
81	9	12
82	9	9
83	1	9
84	1	4
85	1	9

86	0	6
87	1	4
88	4	12

Notes

Output Created		15-MAR-2017 16:49:13
Comments		
Input	Data	C:\Users\Wang chao\Desktop\fig1 b.sav
	Active Dataset	DataSet1
	Filter	<none>
	Weight	<none>
	Split File	<none>
	N of Rows in Working Data File	88
Missing Value Handling	Definition of Missing	User defined missing values are treated as missing.
	Cases Used	Statistics for each analysis are based on the cases with no missing or out-of-range data for any variable in the analysis.
Syntax		T-TEST<br>/TESTVAL=0<br>/MISSING=ANALYSIS<br>/VARIABLES=Peritumor Tumor<br>/CRITERIA=CI(.95).
Resources	Processor Time	00:00:00.00
	Elapsed Time	00:00:00.01

One-Sample Statistics

	N	Mean	Std. Deviation	Std. Error Mean
Peritumor	88	4.02	3.863	.412
Tumor	88	6.26	3.541	.377

One-Sample Test

	Test Value = 0					
					95% Confidence Interval of the Difference	
	t	df	Sig. (2-tailed)	Mean Difference	Lower	Upper

Peritumor	9.770	87	.000	4.023	3.20	4.84
Tumor	16.587	87	.000	6.261	5.51	7.01

Fig. 2G figures and data of the cell cycle in three independent experiments

	NCI-N87-NC			NCI-N87-shPKM2		
G1	64.35%	69.66%	67.72%	72.8%	70.55%	73.42%
S	28.79%	20.6%	24.56%	13.08%	16.05%	10.17%
G2/M	6.85%	9.74%	7.72%	14.12%	13.4%	16.41%

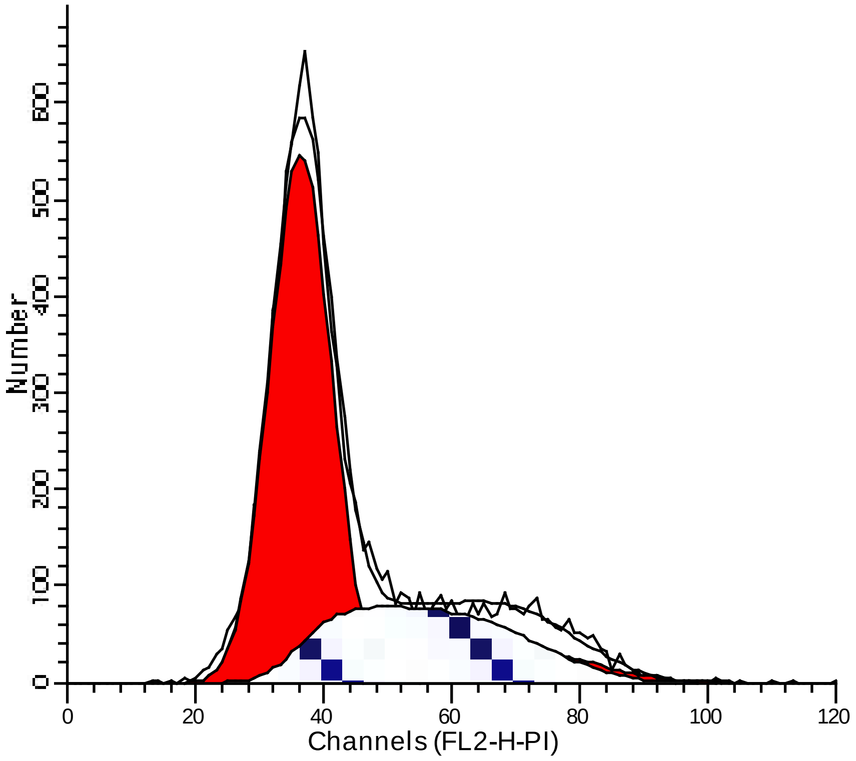

File analyzed: NCI-N87-1
Date analyzed: 12-May-2016
Model: 1nn0n_DSF
Analysis type: Manual analysis

Diploid: 100.00 %
Dip G1: 64.35 % at 36.25
Dip G2: 6.85 % at 73.58
Dip S: 28.79 % G2/G1: 2.03
%CV: 13.15

Total S-Phase: 28.79 %
Total B.A.D.: 0.00 % no debris no aggs

Debris: %
Aggregates: 0.00 %
Modeled events: 10181
All cycle events: 10181

Cycle events per channel: 266
RCS: 1.807

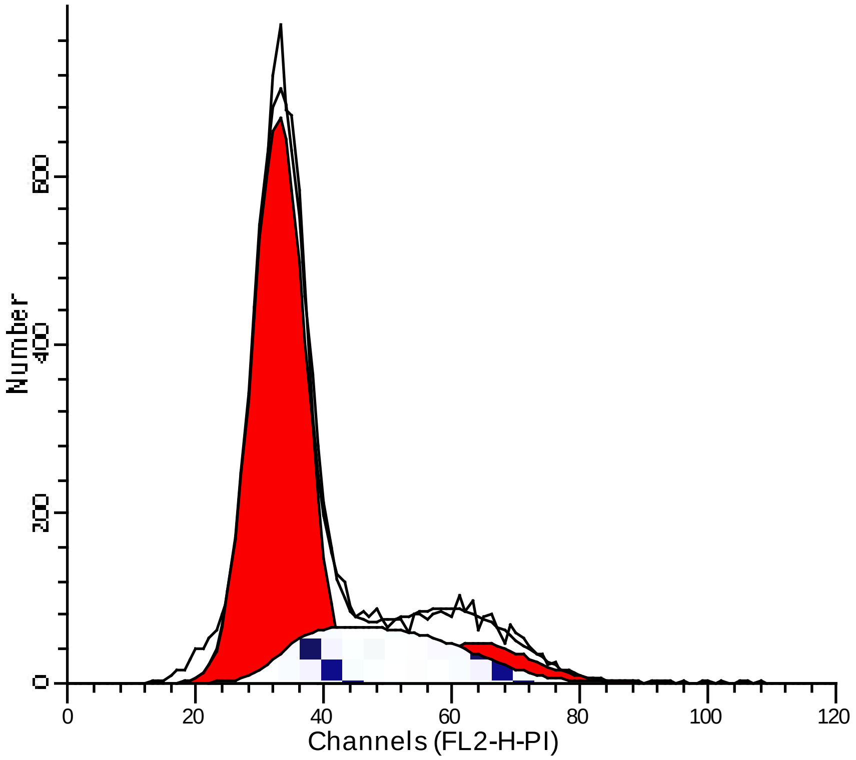

File analyzed: NCI-N87-2
Date analyzed: 12-May-2016
Model: 1nn0n_DSF
Analysis type: Manual analysis

Diploid: 100.00 %
Dip G1: 69.66 % at 32.85
Dip G2: 9.74 % at 63.79
Dip S: 20.60 % G2/G1: 1.94
%CV: 12.51

Total S-Phase: 20.60 %
Total B.A.D.: 0.00 % no debris no aggs

Debris: %
Aggregates: 0.00 %
Modeled events: 9902
All cycle events: 9902
Cycle events per channel: 310
RCS: 2.240

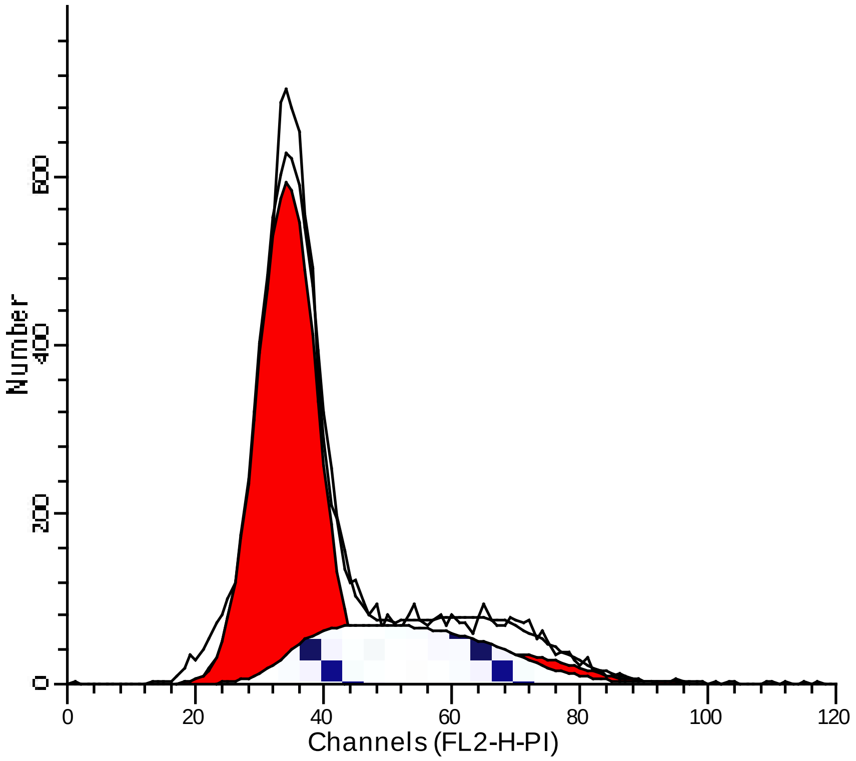

File analyzed: NCI-N87-3
Date analyzed: 12-May-2016
Model: 1nn0n_DSF
Analysis type: Manual analysis

Diploid: 100.00 %
Dip G1: 67.72 % at 34.15
Dip G2: 7.72 % at 69.52
Dip S: 24.56 %   G2/G1: 2.04
%CV: 13.24

Total S-Phase: 24.56 %
Total B.A.D.: 0.00 %   no debris no aggs

Debris: %
Aggregates: 0.00 %
Modeled events: 9947
All cycle events: 9947
Cycle events per channel: 274
RCS: 3.076

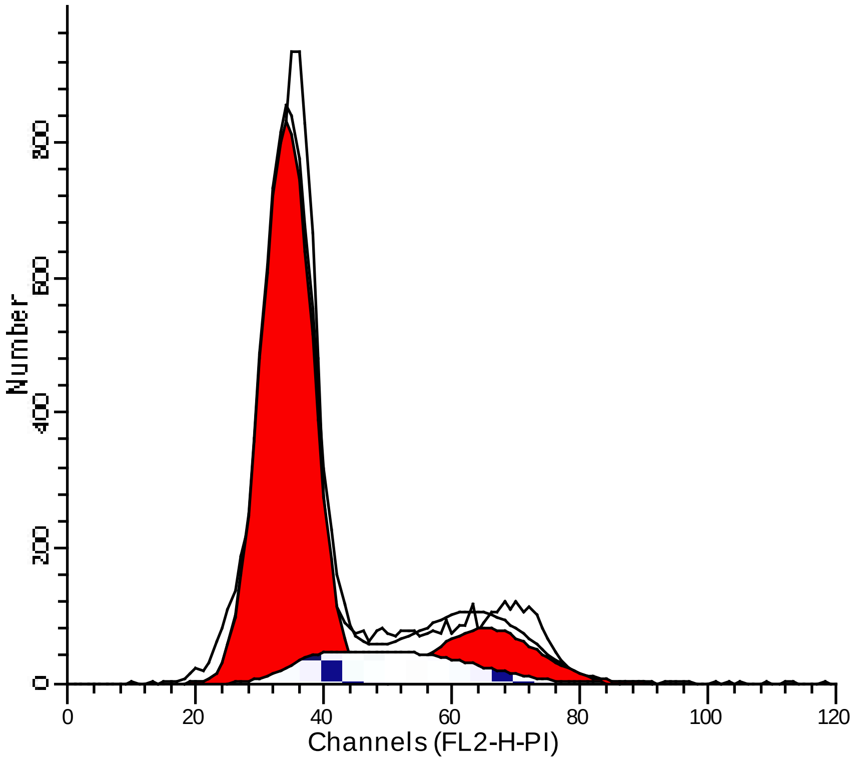

File analyzed: NCI-N87-PKM2-KD-1
Date analyzed: 12-May-2016
Model: 1nn0n_DSF
Analysis type: Manual analysis

Diploid: 100.00 %
    Dip G1: 72.80 % at 34.14
    Dip G2: 14.12 % at 65.13
    Dip S: 13.08 %   G2/G1: 1.91
    %CV: 11.52

Total S-Phase: 13.08 %
Total B.A.D.: 0.00 %   no debris no aggs

Debris: %
Aggregates: 0.00 %
Modeled events: 11307
All cycle events: 11307
Cycle events per channel: 353
RCS: 6.222

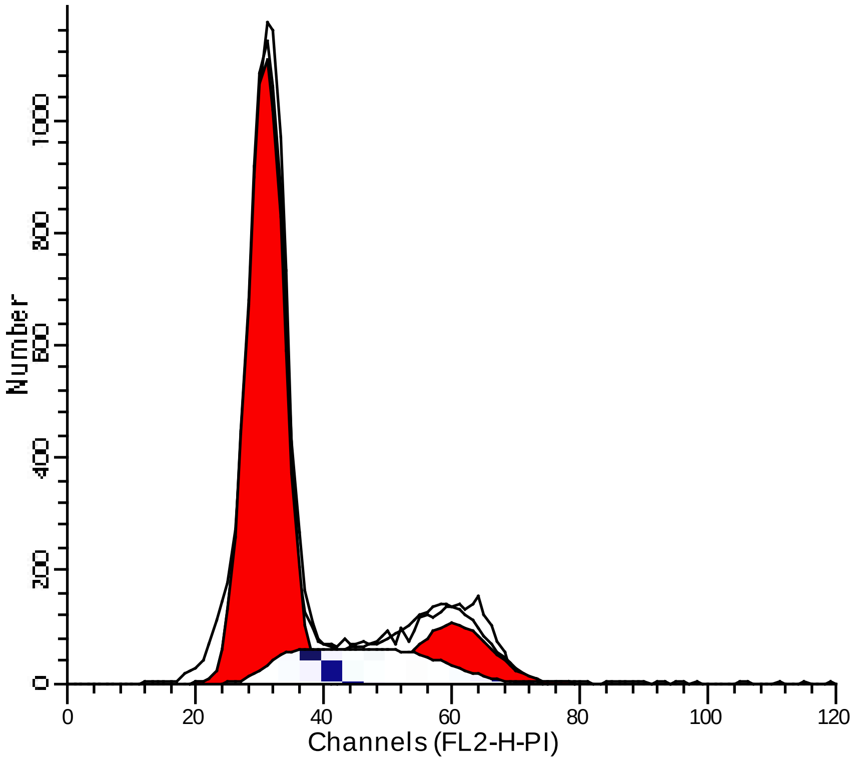

File analyzed: NCI-N87-PKM2-KD-2
Date analyzed: 12-May-2016
Model: 1nn0n_DSF
Analysis type: Manual analysis

Diploid: 100.00 %
Dip G1: 70.55 % at 30.83
Dip G2: 13.40 % at 60.10
Dip S: 16.05 % G2/G1: 1.95
%CV: 9.12

Total S-Phase: 16.05 %
Total B.A.D.: 0.00 % no debris no aggs

Debris: %
Aggregates: 0.00 %
Modeled events: 11134
All cycle events: 11134
Cycle events per channel: 368
RCS: 5.169

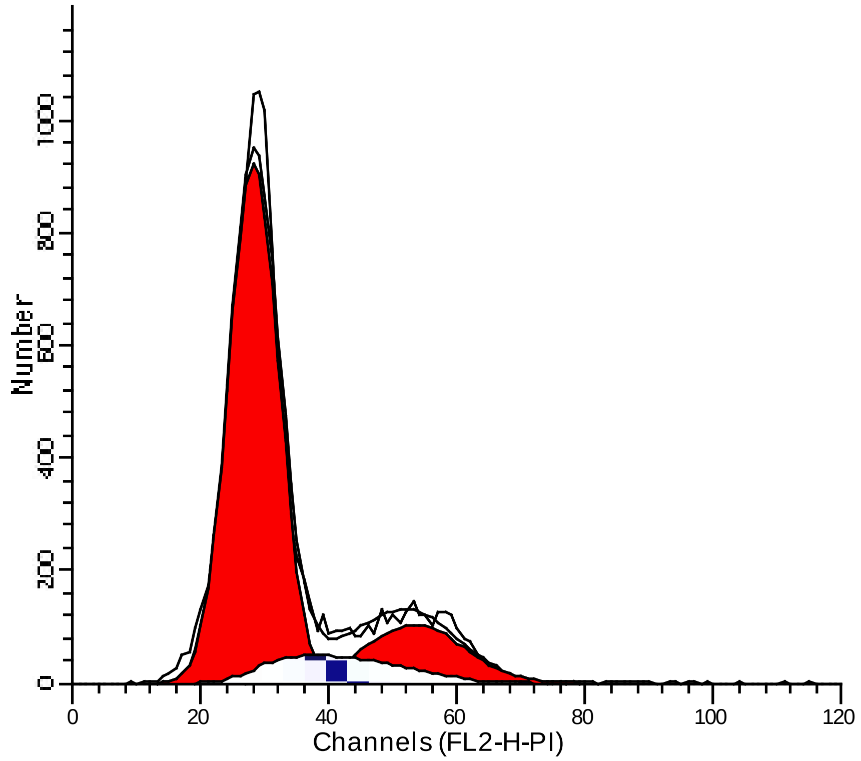

File analyzed: NCI-N87-PKM2-KD-3
Date analyzed: 12-May-2016
Model: 1nn0n_DSF
Analysis type: Manual analysis

Diploid: 100.00 %
Dip G1: 73.42 % at 28.18
Dip G2: 16.41 % at 53.24
Dip S: 10.17 % G2/G1: 1.89
%CV: 13.77

Total S-Phase: 10.17 %
Total B.A.D.: 0.00 % no debris no aggs

Debris: %
Aggregates: 0.00 %
Modeled events: 12289
All cycle events: 12289
Cycle events per channel: 471
RCS: 3.101

Fig. 6I the data of relative band intensity of P-Akt

NCI-N87	NC	KD	NC 3-MA	KD 3-MA
	1.54	1.03	0.69	0.2
	1.5	1.04	0.72	0.22
	1.44	0.9	0.6	0.28

Tukey's multiple comparisons test	Mean Diff.	95% CI of diff.	Significant?	Summary
NC vs. KD	0.5033	0.3472 to 0.6595	Yes	****
NC vs. NC 3-MA	0.8233	0.6672 to 0.9795	Yes	****
NC vs. KD 3-MA	1.260	1.104 to 1.416	Yes	****
KD vs. NC 3-MA	0.3200	0.1638 to 0.4762	Yes	***
KD vs. KD 3-MA	0.7567	0.6005 to 0.9128	Yes	****
NC 3-MA vs. KD 3-MA	0.4367	0.2805 to 0.5928	Yes	****

Fig. S3H the data of relative band intensity of P-Akt

BGC-823	NC	KD	NC 3-MA	KD 3-MA
	1.01	0.86	0.8	0.35
	1.18	0.83	0.78	0.32
	1.23	0.8	0.75	0.3

Tukey's multiple comparisons test	Mean Diff.	95% CI of diff.	Significant?	Summary
NC vs. KD	0.3120	0.1486 to 0.4754	Yes	**
NC vs. NC 3-MA	0.3616	0.1982 to 0.5250	Yes	***
NC vs. KD 3-MA	0.8152	0.6518 to 0.9787	Yes	****
KD vs. NC 3-MA	0.04960	-0.1138 to 0.2130	No	ns
KD vs. KD 3-MA	0.5032	0.3398 to 0.6667	Yes	****
NC 3-MA vs. KD 3-MA	0.4536	0.2902 to 0.6171	Yes	****

* P<0.05. ** P<0.01. *** P<0.001. **** P<0.0001

Supplementary files 2

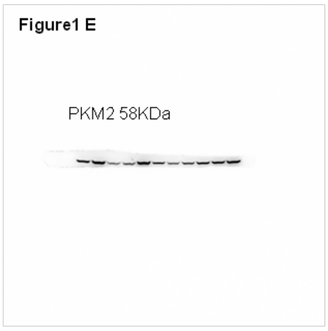

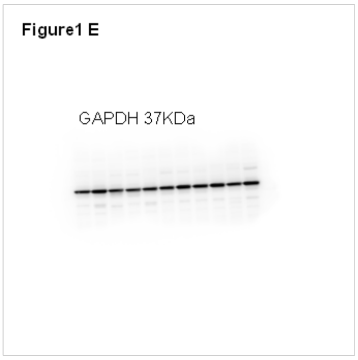

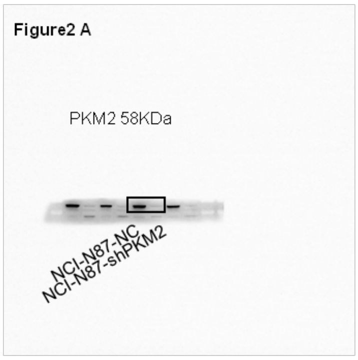

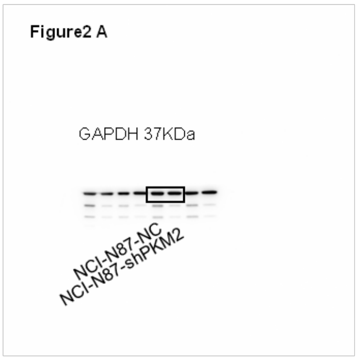

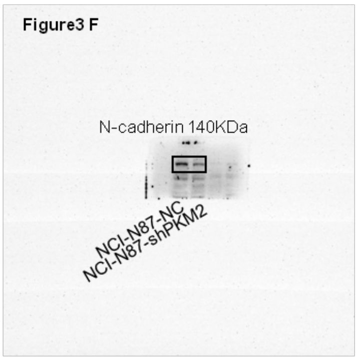

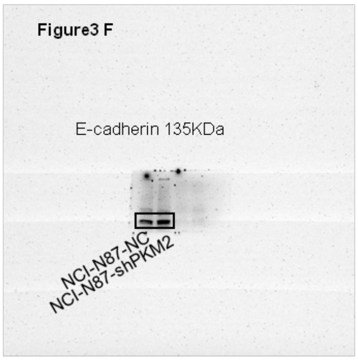

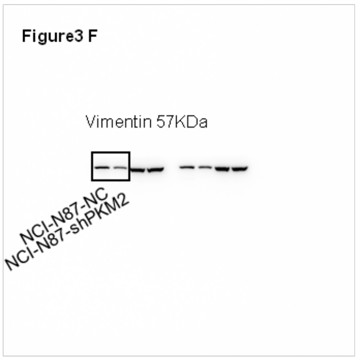

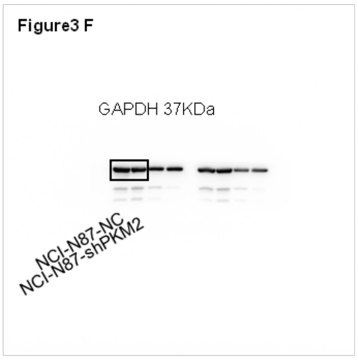

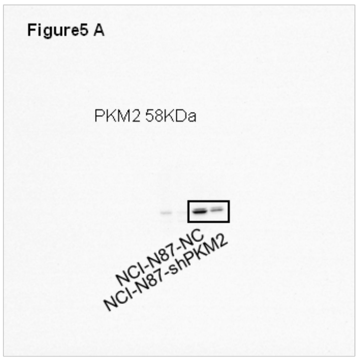

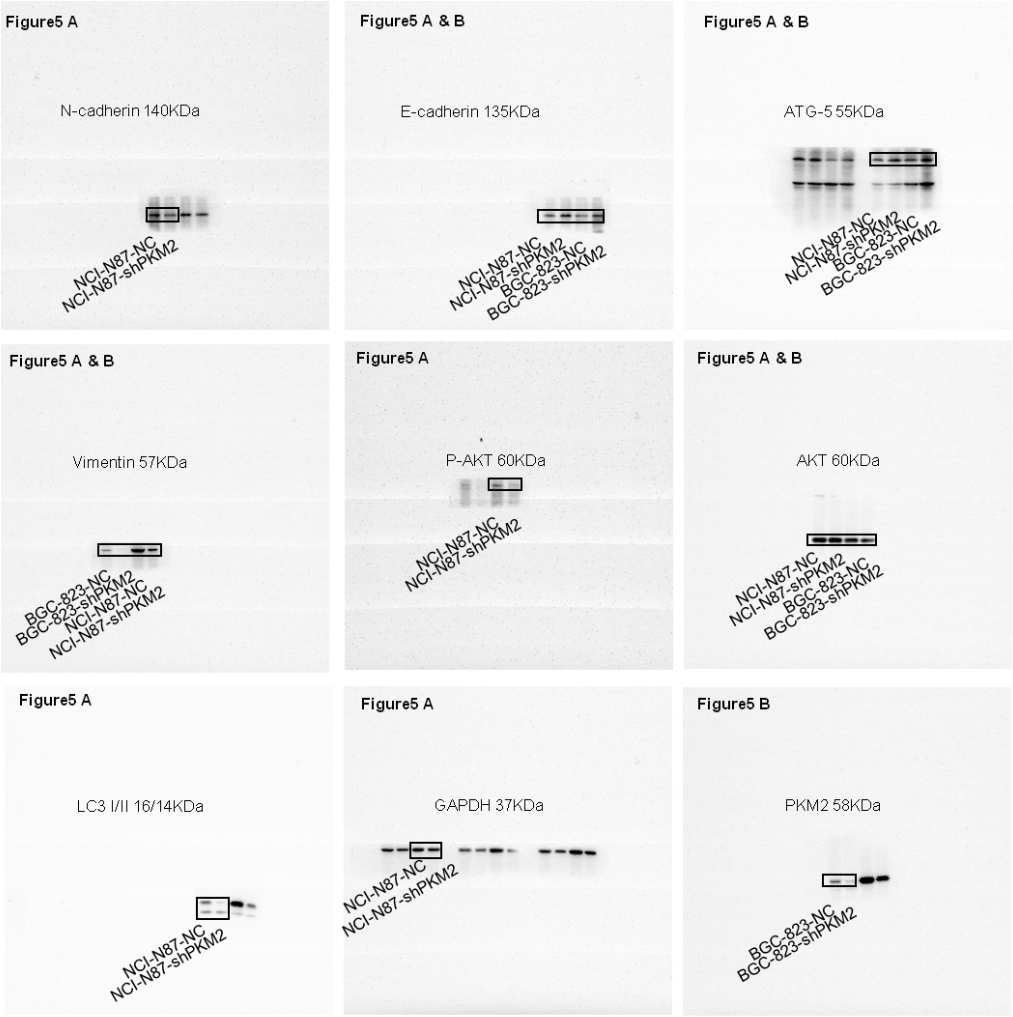
Figure5 A
N-cadherin 140KDa
NCI-N87-NC
NCI-N87-shPKM2
Figure5 A & B
E-cadherin 135KDa
NCI-N87-NC
NCI-N87-shPKM2
BGC-823-NC
BGC-823-shPKM2
Figure5 A & B
ATG-5 55KDa
NCI-N87-NC
NCI-N87-shPKM2
BGC-823-NC
BGC-823-shPKM2
Figure5 A & B
Vimentin 57KDa
BGC-823-NC
BGC-823-shPKM2
NCI-N87-NC
NCI-N87-shPKM2
Figure5 A
P-AKT 60KDa
NCI-N87-NC
NCI-N87-shPKM2
Figure5 A & B
AKT 60KDa
NCI-N87-NC
NCI-N87-shPKM2
BGC-823-NC
BGC-823-shPKM2
Figure5 A
LC3 I/II 16/14KDa
NCI-N87-NC
NCI-N87-shPKM2
Figure5 A
GAPDH 37KDa
NCI-N87-NC
NCI-N87-shPKM2
Figure5 B
PKM2 58KDa
BGC-823-NC
BGC-823-shPKM2

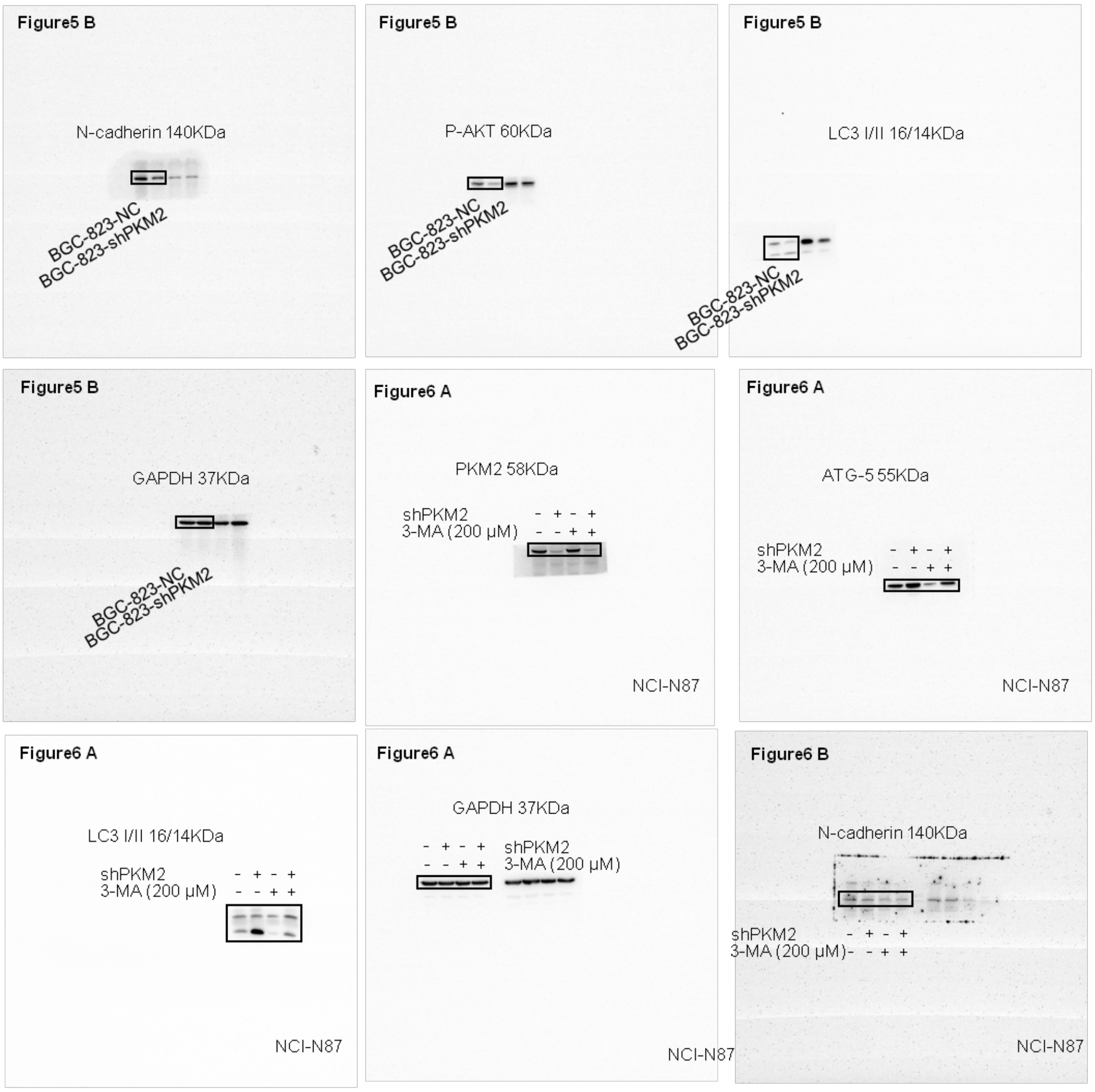
Figure5 B
N-cadherin 140KDa
BGC-823-NC
BGC-823-shPKM2
Figure5 B
P-AKT 60KDa
BGC-823-NC
BGC-823-shPKM2
Figure5 B
LC3 I/II 16/14KDa
BGC-823-NC
BGC-823-shPKM2
Figure5 B
GAPDH 37KDa
BGC-823-NC
BGC-823-shPKM2
Figure6 A
PKM2 58KDa
shPKM2 - + - +
3-MA (200 μM) - - + +
NCI-N87
Figure6 A
ATG-5 55KDa
shPKM2 - + - +
3-MA (200 μM) - - + +
NCI-N87
Figure6 A
LC3 I/II 16/14KDa
shPKM2 - + - +
3-MA (200 μM) - - + +
NCI-N87
Figure6 A
GAPDH 37KDa
- + - +
- - + +
shPKM2
3-MA (200 μM)
NCI-N87
Figure6 B
N-cadherin 140KDa
shPKM2 - + - +
3-MA (200 μM)- - + +
NCI-N87

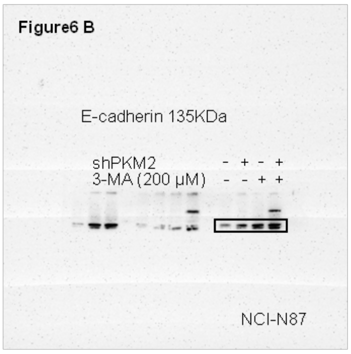
Figure6 B
E-cadherin 135KDa
shPKM2 - + - +
3-MA (200 μM) - - + +
NCI-N87

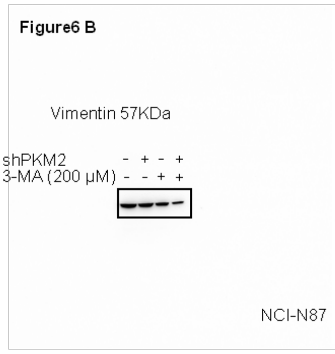
Figure6 B
Vimentin 57KDa
shPKM2 - + - +
3-MA (200 μM) - - + +
NCI-N87

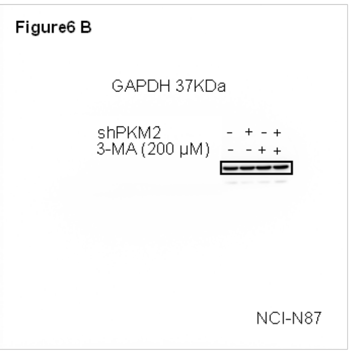
Figure6 B
GAPDH 37KDa
shPKM2 - + - +
3-MA (200 μM) - - + +
NCI-N87

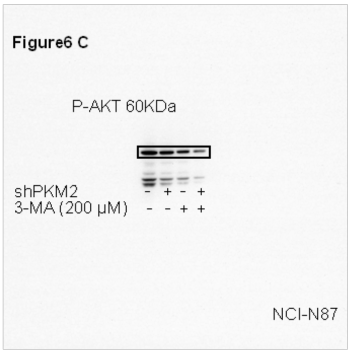
Figure6 C
P-AKT 60KDa
shPKM2 - + - +
3-MA (200 μM) - - + +
NCI-N87

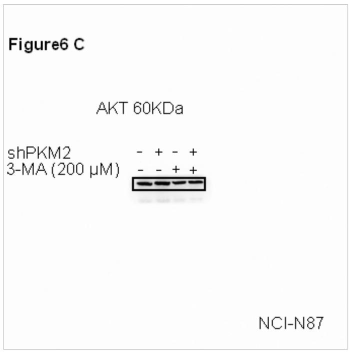
Figure6 C
AKT 60KDa
shPKM2 - + - +
3-MA (200 μM) - - + +
NCI-N87

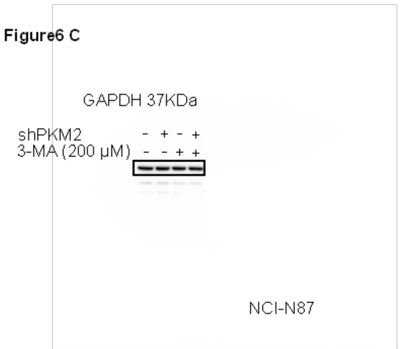
Figure6 C
GAPDH 37KDa
shPKM2 - + - +
3-MA (200 μM) - - + +
NCI-N87

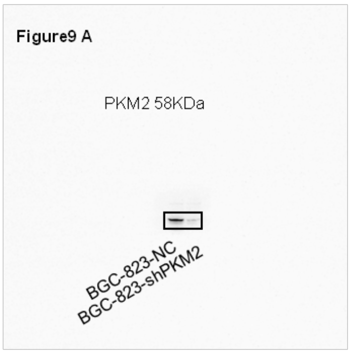
Figure9 A
PKM2 58KDa
BGC-823-NC
BGC-823-shPKM2

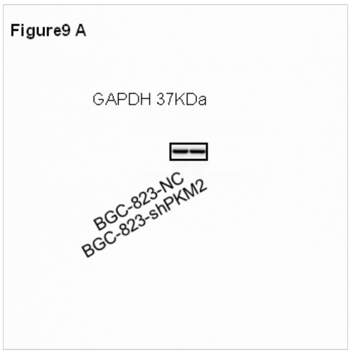
Figure9 A
GAPDH 37KDa
BGC-823-NC
BGC-823-shPKM2

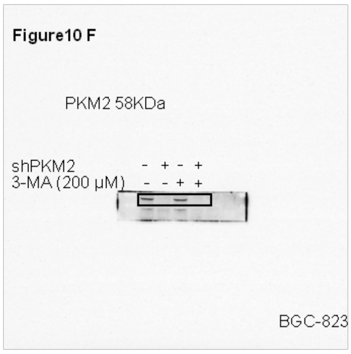
Figure10 F
PKM2 58KDa
shPKM2 - + - +
3-MA (200 μM) - - + +
BGC-823

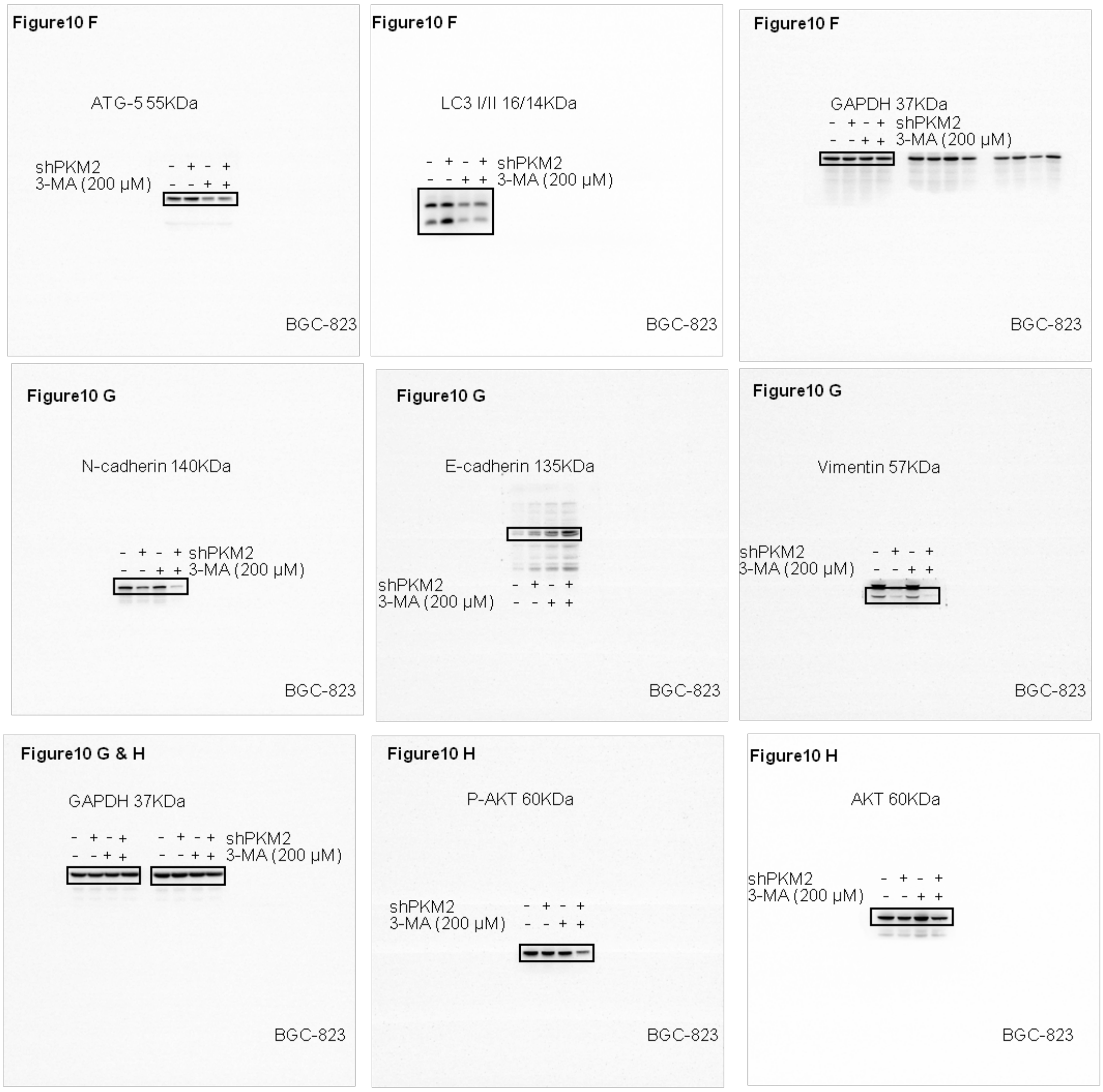
Figure10 F
ATG-5 55KDa
shPKM2 - + - +
3-MA (200 μM) - - + +
BGC-823
Figure10 F
LC3 I/II 16/14KDa
- + - + shPKM2
- - + + 3-MA (200 μM)
BGC-823
Figure10 F
GAPDH 37KDa
- + - + shPKM2
- - + + 3-MA (200 μM)
BGC-823
Figure10 G
N-cadherin 140KDa
- + - + shPKM2
- - + + 3-MA (200 μM)
BGC-823
Figure10 G
E-cadherin 135KDa
shPKM2 - + - +
3-MA (200 μM) - - + +
BGC-823
Figure10 G
Vimentin 57KDa
shPKM2 - + - +
3-MA (200 μM) - - + +
BGC-823
Figure10 G & H
GAPDH 37KDa
- + - + - + - + shPKM2
- - + + - - + + 3-MA (200 μM)
BGC-823
Figure10 H
P-AKT 60KDa
shPKM2 - + - +
3-MA (200 μM) - - + +
BGC-823
Figure10 H
AKT 60KDa
shPKM2 - + - +
3-MA (200 μM) - - + +
BGC-823
